# Mucosal Vaccination for Influenza Protection Enhanced by Catalytic Immune‐Adjuvant

**DOI:** 10.1002/advs.202000771

**Published:** 2020-08-02

**Authors:** Tao Qin, Shang Ma, Xinyu Miao, Yan Tang, Dandan Huangfu, Jinyuan Wang, Jing Jiang, Nuo Xu, Yuncong Yin, Sujuan Chen, Xiufan Liu, Yinyan Yin, Daxin Peng, Lizeng Gao

**Affiliations:** ^1^ College of Veterinary Medicine Yangzhou University Yangzhou Jiangsu 225009 P. R. China; ^2^ Institute for Translational Medicine, School of Medicine Yangzhou University Yangzhou Jiangsu 225009 P. R. China; ^3^ CAS Engineering Laboratory for Nanozyme Institute of Biophysics Chinese Academy of Sciences Beijing 100101 P. R. China; ^4^ Jiangsu Co‐Innovation Center for the Prevention and Control of Important Animal Infectious Disease and Zoonoses Yangzhou Jiangsu 225009 P. R. China; ^5^ Joint Laboratory Safety of International Cooperation of Agriculture & Agricultural‐Products Yangzhou Jiangsu 225009 P. R. China; ^6^ Jiangsu Research Centre of Engineering and Technology for Prevention and Control of Poultry Disease Yangzhou 225009 P. R. China

**Keywords:** adjuvant, influenza virus, mucosal vaccine, nanozyme

## Abstract

Influenza poses a severe threat to global health. Despite the whole inactivated virus (WIV)‐based nasal vaccine being a promising strategy for influenza protection, the mucosal barrier is still a bottleneck of the nasal vaccine. Here, a catalytic mucosal adjuvant strategy for an influenza WIV nasal vaccine based on chitosan (CS) functionalized iron oxide nanozyme (IONzyme) is developed. The results reveal that CS‐IONzyme increases antigen adhesion to nasal mucosa by 30‐fold compared to H1N1 WIV alone. Next, CS‐IONzyme facilitates H1N1 WIV to enhance CCL20‐driven submucosal dendritic cell (DC) recruitment and transepithelial dendrite(TED) formation for viral uptake via the toll‐like receptor(TLR) 2/4‐dependent pathway. Moreover, IONzyme with enhanced peroxidase (POD)‐like activity by CS modification catalyzes a reactive oxygen species (ROS)‐dependent DC maturation, which further enhances the migration of H1N1 WIV‐loaded DCs into the draining lymph nodes for antigen presentation. Finally, CS‐IONzyme‐based nasal vaccine triggers an 8.9‐fold increase of IgA‐mucosal adaptive immunity in mice, which provides a 100% protection against influenza, while only a 30% protection by H1N1 WIV alone. This work provides an antiviral alternative for designing nasal vaccines based on IONzyme to combat influenza infection.

## Graphical abstract

A CS‐IONzyme‐based influenza mucosal vaccine with dual targeting of both nasal mucosa and submucosal DCs, leading to a robust immunoprotection against influenza virus is designed. Utilizing an excellent POD‐like activity, a catalytic immune‐adjuvant CS‐IONzyme against the bottleneck of mucosal vaccines is successfully developed by both fully mobilizing submucosal DCs to form TEDs for antigen uptake and strongly activating DC maturation.

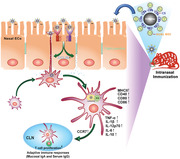

## 1 Introduction

Influenza is a highly contagious disease that causes high morbidity and mortality worldwide each year. To date, the influenza A (H1N1) pdm09 virus which first emerged in 2009 and resulted in ≈575 400 worldwide deaths.^[^ ^1^ ^]^ Furthermore, H5N1 and H7N9 highly pathogenic avian influenza (HPAI) viruses caused nearly 53% and 39% human case fatality rate, respectively.^[^ ^2^ ^]^ The nasal cavity is the primary entry site of influenza virus, and the viral infection can be effectively discontinued in the first time if mucosal immunity is well established.^[^ ^3^ ^]^ Despite the live attenuated intranasal influenza vaccines posing a limited infection to nasal mucosa can strongly induce the effective humoral and cellular immunity, potential safety risks of administering a live virus into a population before the target strain are widespread.^[^ ^4^ ^]^ The whole inactivated virus (WIV) vaccine given via intranasal immunization is a greatly potential strategy, basing on its needle‐free delivery, high security against virus recombination, high mucosal antibody levels at the pathogen's entry site against viral invading and transmission, and good cross‐protection against various strains.^[^ ^5^ ^]^ Nonetheless, the efficacy of intranasal immunization is currently poor, primarily because of a series of nasal barriers, including mucus, cilia, and compact epithelium, which block antigens delivery to submucosal antigen presenting cells (APCs)‐dendritic cells (DCs) and limit subsequent DC maturation levels for initiating protective adaptive immunity.^[^ ^6^ ^]^

Immune‐adjuvants can excellently increase WIV vaccine efficacy by modeling the quality and the quantity of the host immune responses. Notably, they can boost the rate, magnitude, or format of the immune responses. Traditional adjuvants are mainly considered to how to stimulate the immune system, such as aluminum salt, toll‐like receptor (TLR) agonist, cytokines, et al.^[^ ^7^ ^]^ However, most adjuvants have good efficacies when using for the injection immunization but not for mucosal immunization. Cholera toxin (CT) is an ideal mucosal adjuvant,^[^ ^8^ ^]^ however, the toxicity with high risk limits their clinical applications.^[^ ^9^ ^]^ Chitosan, also known as *β* (1‐4)‐linked 2‐acetamido‐2‐deoxy‐*β*‐Dglucose (N‐acetyl glucosamine), is a cationic polysaccharide, which is a promising vehicle for mucosal vaccine delivery due to its biodegradability, biocompatibility, and mucoadhesion in nature, but the insolubility and the aggregation are the key barriers for biomedical applications in spite of various artificial modifications by physical and chemical methods.^[^ ^10^ ^]^ Therefore, mucosal adjuvants with multiple functions, including mucosal barrier crossing, immune targeting, biosafety, and low‐cost for influenza mucosal vaccines are needed to develop.

Iron oxide nanoparticles have been widely used in biomedical fields due to their excellent magnetic properties, biocompatibility, biosafety, and biodegradability.^[^ ^11^ ^]^ In 2007, the finding that Fe_3_O_4_ nanoparticles posed intrinsic peroxidase (POD) ‐like activity,^[^ ^12^ ^]^ which broke the traditional concept that inorganic materials were bio‐inert and opened up a new horizon for nanomaterials. In the last decade, hundreds of nanomaterials have been found to perform intrinsic enzyme‐like activities, which have been uniformly denoted as nanozymes.^[^ ^13^ ^]^ In comparison to nature enzymes, nanozyme activities can be regulated by modulating the size, shape, dopant and surface charge.^[^ ^14^ ^]^ Iron oxide (Fe_3_O_4_) nanoparticles, as the most typical nanozymes, perform multiple enzyme‐like activities, including peroxidase, catalase, and lipoxidase‐like activities, which are thus referred to iron oxide nanozymes (IONzymes).^[^ ^11^, ^15^ ^]^ These catalytic properties are consequently applied to develop immunological diagnostic methods,^[^ ^16^ ^]^ anti‐cancer,^[^ ^17^ ^]^ and anti‐pathogens.^[^ ^15^, ^18^ ^]^ Interestingly, IONzyme poses a catalase‐like activity at neutral pH conditions, which is conducive to scavenge reactive oxygen species (ROS). In contrast, at acidic pH conditions, IONzyme exhibits a POD‐like activity, which is beneficial to the generation of ROS.^[^ ^11^, ^19^ ^]^ In the mucosal immune system, antigens are mainly delivered to the acidic lysosomes of submucosal DCs for processing and presentation.^[^ ^20^ ^]^ Subsequently, the effectiveness of downstream immunity is mainly influenced by DC maturation,^[^ ^21^ ^]^ which can be regulated by intracellular ROS levels.^[^ ^22^ ^]^ On the basis of these earlier studies, it drives us to hypothesize that IONzyme may provide a possibility to develop a novel catalytic adjuvant for mucosal vaccine by utilizing its POD‐like activity at the acidic lysosomes to target DC maturation.

**Scheme 1:**
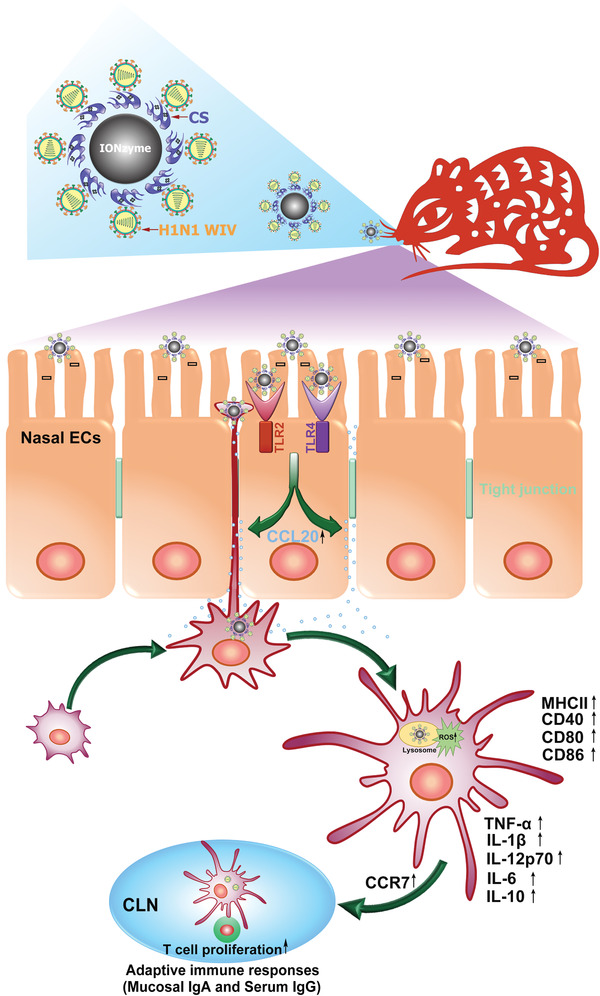
Schematic of proposed mechanism for enhancing antigen‐specific immune response by the CS‐IONzyme‐based influenza vaccine. CS‐IONzyme first enhances the adhesion levels of H1N1 WIV to nasal mucosa after intranasal immunization of CS‐IONzyme‐based influenza vaccine in mice. Next, CS‐IONzyme strongly stimulates nasal epithelial cells (ECs) to secrete chemokine CCL20 via toll‐like receptor (TLR) 2/4 pathway, which recruits more DCs into the submucosal regions to form transepithelial dendrites (TEDs) for H1N1 WIV uptake. After viral capture, DCs gradually reach the phenotypic and functional maturation and then quickly migrate into draining cervical lymph nodes (CLNs) for antigen presentation. Finally, the antigen‐specific immune response is initiated.

Here, we present a successful paradigm of nasal mucosal delivery vaccine for H1N1 WIV basing on chitosan (CS) functionalized IONzyme, which provides a strong protective immunity against influenza virus with a lethal attack. Surprisingly, CS modification not only sharply enhances the adhesive capacity of antigens to nasal mucosa, but also strengthens the POD‐like activity of IONzyme for ROS‐dependent DC maturation (**Scheme** **1**), thus implying a promising strategy for designing of mucosal vaccines against influenza.

## 2 Results

### 2.1 CS‐IONzyme Improves the Mucosal Adhesion of H1N1 WIV

**Figure 1:**
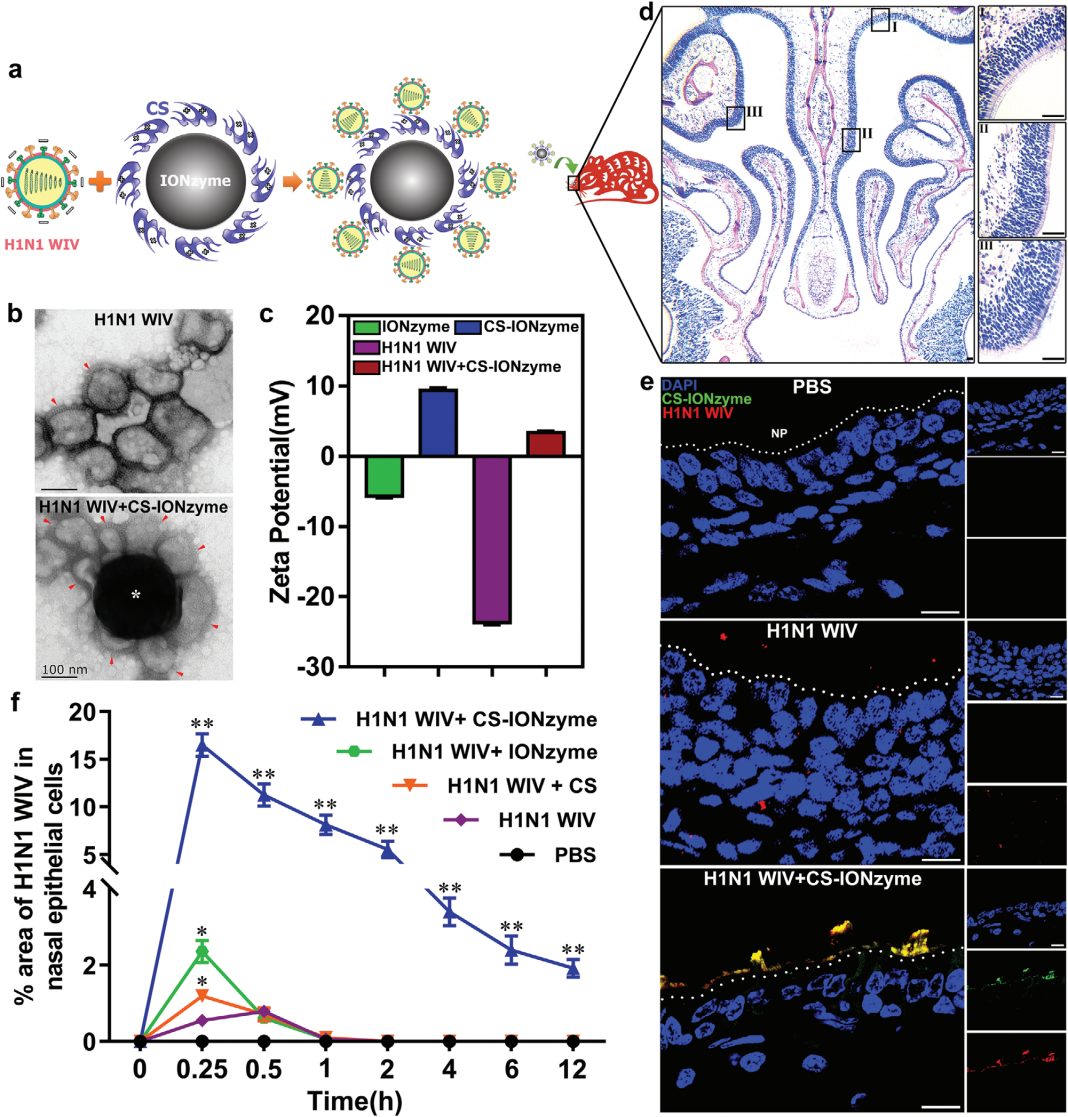
Vaccine design and antigen adhesion to nasal mucosa. a) Schematic demonstrating a design of mucosal delivery vaccine by combination of CS‐IONzyme and H1N1 WIV. b) TEM observation of a complex of CS‐IONzyme (*) and H1N1 WIV (red arrows). c) Zeta potential detection by Malvern Instrument Nano‐ ES90. d,e) Mice (*n* = 6 per group) were nasally administered with either PBS or H1N1 WIV or CS‐IONzyme and H1N1 WIV complexes for 0.5 h and noses were collected. d) Cross‐sections using H&E staining revealed the histological characteristic of the murine nasal tissues. Enlargements of the regions in the black frames (I–III) show pseudostratified columnar ciliated epitheliums located in the nasal mucosa. e) Frozen sections of nasal tissues were observed for evaluating the adhesion levels of H1N1 WIV to nasal mucosa by using CLSM. CS‐IONzyme (FITC; green), H1N1 WIV (DyLight 550; red), and Nuclei (4′, 6‐diamidino‐2‐phenylindole (DAPI); blue). f) Mice were nasally immunized with either PBS or various vaccines for 0.25, 0.5, 1, 2, 4, 6, and 12 h, and then noses were collected for frozen sections. Quantifications of the H1N1 WIV‐positive areas in the nasal ECs. Results are expressed as percentage of area occupied by positive H1N1 WIV compared with that of nasal ECs. The value obtained from ten random images of six mice. Data shown are the means ± s.d. Statistical significance is assessed by unpaired Student's two‐sided *t*‐test to compare the group of H1N1 WIV. *P < 0.05; **P < 0.01. Results are from one representative experiment of three performed. Bars: (b) 100 nm; (d) 50 µm; (e) 10 µm.

We designed a novel mucosal delivery vaccine by combination of CS‐IONzyme and H1N1 WIV (**Figure** **1**a). First, CS‐IONzyme was synthesized by using a solvothermal method, where CS in the reaction system acted both as a ligand and a surface functionalization agent. Three kinds of chitosan (low (50–190 KDa), medium (190–310 KDa), and high (310–375 KDa) molecular weight) functionalized IONzyme (named CS‐IONzyme) were spheres of ≈250 nm in diameter, which were a bit bigger than IONzyme (Figure 1b and Figure S1a,b, Supporting Information). Moreover, IONzyme with CS (low molecular weight) was chosen for the following research because it exhibited a high POD‐like activity as demonstrated by the colorimetric reaction of hydrogen peroxide (H_2_O_2_) (Figure S1c, Supporting Information). Thermogravimetric analysis suggested that the weight ratio of the modifier on CS (50–190 KDa)‐IONzyme was 8.5% (Figure S1d, Supporting Information). X‐ray photoelectron spectroscopy (XPS) verified that, besides the peaks of Fe 2p, O 1s and C 1s orbitals at 710, 529, and 284 eV, CS‐modified IONzyme also had a peak of N 1s orbital at 399 eV (Figure S1e, Supporting Information). The zeta potential measurement indicated that positive charges were introduced to the surface of IONzyme because of CS modification (Figure 1c). These results suggested that CS can be steadily modified onto the IONzyme, which not only strengthened the POD‐like activity but also altered the surface charge of IONzyme for potential antigen delivery. The bio‐stability assay indicated that CS‐IONzyme was stable in the simulated body fluid (SBF) and cell culture medium (EMEM) completed with 10% of fetal bovine serum without morphological changes by transmission electron microscope (TEM) and scanning electron microscope (SEM) observing (Figure S2a,b, Supporting Information). The POD‐like activity of CS‐IONzyme did not display a sharp decrease before 3 d, which is a key stage for antigen presentation and the innate immune response activation of DCs (Figure S2c, Supporting Information).

Furthermore, basing on electrostatic interactions between the positive surface charges on CS‐IONzyme and the negative surface charges on H1N1 WIV, we mixed CS‐IONzyme and H1N1 WIV to prepare CS‐IONzyme and H1N1 WIV complexes. The TEM image showed 7–8 virions cinctured and tightly combined with CS‐IONzyme particles (Figure 1b). The zeta potential data indicated that CS‐IONzyme and H1N1 WIV complex showed positive charges (Figure 1c), implying that it was possible to develop an influenza vaccine through a charge attraction to promote antigen delivery to the nasal mucosa (negative charges). First, the biosafety of IONzyme and CS‐IONzyme was evaluated in mice by intranasal immunization. The body weight was monitored and there was no change in both IONzyme and CS‐IONzyme group compared with PBS group even if the dose was used up to 5 mg kg^−1^ (Figure S3a, Supporting Information). Hematoxylin and eosin (H&E) staining of nasal cavity display no pathological changes (Figure S3b,c, Supporting Information). In vitro, the cytotoxicity test in the A549 respiratory epithelial cells and murine DCs indicated that CS‐IONzyme (≤300 µg mL^−1^) did not affect the cellular viability (Figure S3d,e, Supporting Information). These data suggested that CS‐IONzyme had a good biosafety. Moreover, the mice were intranasally immunized with CS‐IONzyme and H1N1 WIV complexes, the viral adhesion levels to nasal mucosa were determined. Nasal cavity tissues were then collected, and the H&E staining images showed that nasal mucosa was composed of pseudostratified ciliated columnar epithelium (Figure 1d), which is a key obstacle against H1N1 WIV adhesion to nasal mucosa (Figure 1e). Of note, confocal laser scanning microscopy (CLSM) image displayed a strong colocalization between CS‐IONzyme and H1N1 WIV (Figure 1e), suggesting that a stable complex was surely formed even if in vivo. Surprisingly, the amount of CS‐IONzyme‐H1N1 WIV complex was remarkably increased up to about 30‐fold compared to that of H1N1 WIV at 15 min. Additionally, our complexes can steadily adhere to the surface of mucosa even if at 12 h, however, the viral particles in H1N1 WIV alone, H1N1 WIV plus CS, and H1N1 WIV plus IONzyme groups were hardly detected after 1 h (Figure 1f and Figure S4, Supporting Information). Together, these results strongly suggested that CS‐IONzyme and H1N1 WIV complexes possessed an effective adhesion ability against mucociliary clearance.

### 2.2 CS‐IONzyme Raises DC Recruitment and TED Formation via CCL20

**Figure 2:**
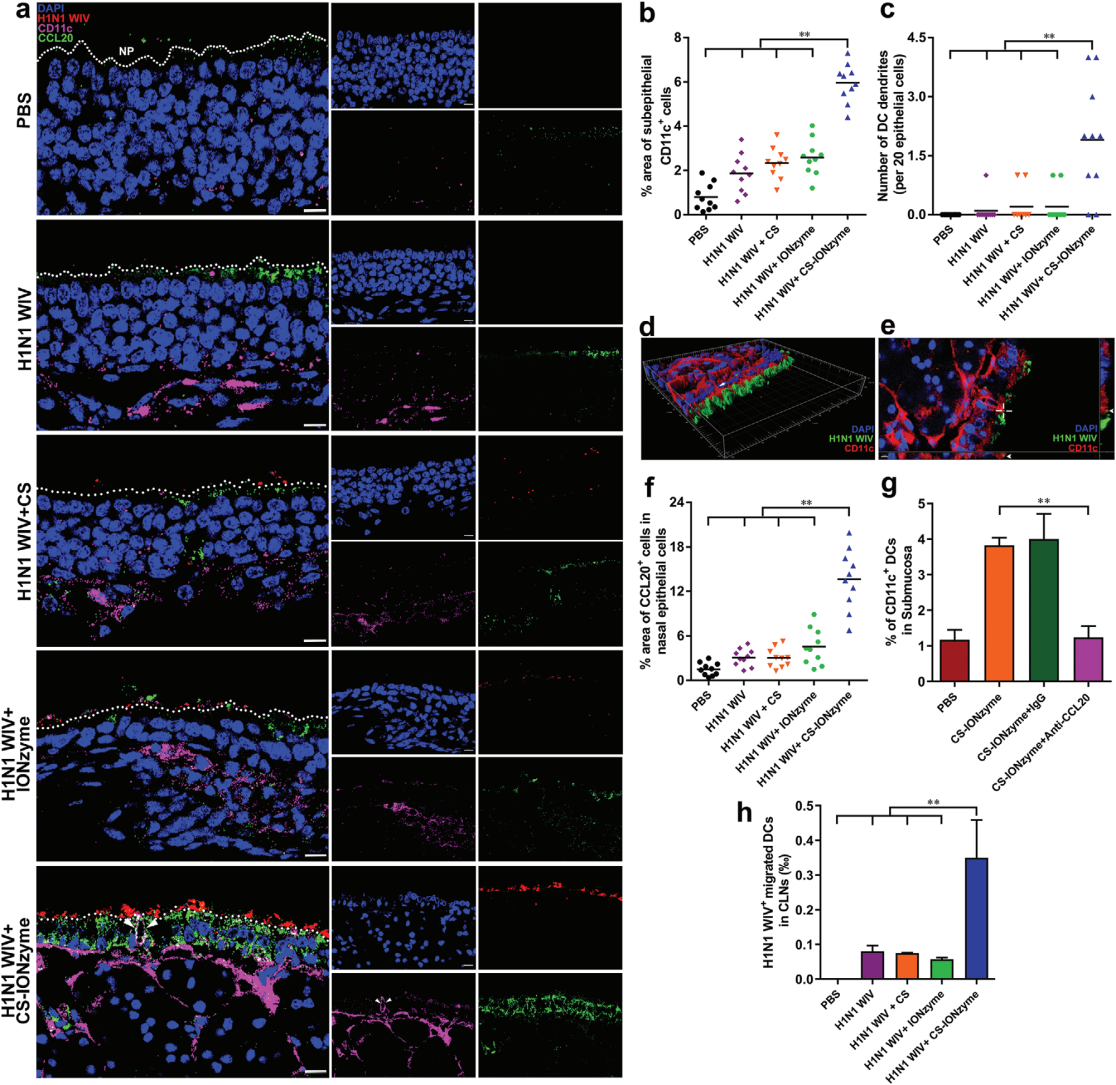
CCL20‐droved DC recruitment and TED formation in nasal mucosa. a) Immunofluorescence stain of the murine nasal cavity (*n* = 10 per group) after intranasally administering of PBS, H1N1 WIV, H1N1 WIV plus CS, H1N1 WIV plus IONzyme, or CS‐IONzyme and H1N1 WIV complexes for 0.5 h. Nuclei (DAPI; blue), H1N1 WIV (DyLight 550; red), CD11c (Alexa Fluor 649; pink), CCL20 (Alexa Fluor 488; green), NP, nasal passage. TEDs are indicated by arrows. b,c) Quantifications of the positive CD11c cells or TEDs number are show in scatter plots. The results of CD11c^+^ cells are expressed as the percentage of the area occupied by positive cells compared to that of lamina propria. The number of TEDs is also quantified, and the values represent the number of TEDs per 20 ECs. Each dot represents the value obtained from one field of ten random fields from ten mice. Horizontal lines across the scatter diagram represent mean values. d,e) 3D rendering (d) or cross‐sectional images (e) of representative fields obtained with Imaris 7.2 software, showing virus uptake (triangles or cross hairs) by TEDs (long arrow). Nuclei (DAPI; blue), H1N1 WIV (DyLight 488; green), CD11c (Alexa Fluor 649; red). f) Quantifications of the positive CCL20 cells are show in scatter plots. The results of CCL20^+^ cells are expressed as the percentage of the area occupied by positive cells compared to that of lamina propria. Each dot represents the value obtained from one field of ten random fields from ten mice. Horizontal lines across the scatter diagram represent mean values. g) CCL20 neutralizing antibody (100 µg per mouse), or rabbit IgG control was administered to mice (*n* = 6 per group) intraperitoneally for 2 h, and then CS‐IONzyme was intranasally dropped in the mice for 0.5 h. The individual cells were isolated from nasal cavity without nasal‐associated lymphoid tissues (NALTs) for FCM analysis. Quantifications of the DCs (CD11c^+^) that migrated into the submucosa. h) Mice (*n* = 6 per group) were nasally dropped CS‐IONzyme and H1N1 WIV complexes and other different control groups for 2 h. CLN cells were isolated and gated by FCM. Quantifications of the H1N1 WIV‐loaded DCs (CD11c^+^CCR7^+^DY488‐H1N1 WIV^+^) that migrated into the CLNs. Results are from one representative experiment of two performed. All of data shown are the means ± s.d. Statistical significance is assessed by One‐way ANOVA for multiple comparisons. *P < 0.05; **P < 0.01. Bars: (a) 10 µm; (e) 5 µm.

Submucosal DCs as “‘sentries” patrol under the epithelium and use their transepithelial dendrite (TED) to monitor luminal “‘antigens.”^[^ ^23^ ^]^ Therefore, whether many submucosal DCs can be mobilized, which is a pivotal step for initiating the downstream innate and adaptive immune response. To investigate whether CS‐IONzyme has a capability to assist H1N1 WIV in recruiting submucosal DCs and TED formation, we performed CLSM on murine nasal tissues after intranasal immunization of CS‐IONzyme and H1N1 WIV complexes. Immunofluorescence images showed that abundant DCs were recruited into submucosal lamina propria in the group of CS‐IONzyme and H1N1 WIV complexes. Notably, a long dendrite extended by submucosal DCs can be observed between two epithelial cells (arrows), indicating that TEDs were formed for antigen uptake (**Figure** **2**a). Quantification results showed that the number of recruited DCs and formed TEDs induced by CS‐IONzyme and H1N1 WIV complexes was significantly increased by approximately threefold and 19‐fold, respectively, when compared with H1N1 WIV alone (Figure 2b,c). To further determine that if submucosal DCs are involved in transepithelial uptake of luminal H1N1 WIV, we performed CLSM with the 3D rendering or cross‐sectional observation mode. We observed that the recruited DCs were able to use their TEDs to sample the luminal fluorescent‐labeled viruses and transport them to the body of DCs for antigen presentation (Figure 2d,e and Figure S5, Supporting Information). Meanwhile, Flow cytometry (FCM) results also showed that the number of H1N1 WIV‐loaded CD11c^+^ DCs located in the submucosa was robustly increased by approximately tenfold in the group of CS‐IONzyme and H1N1 WIV complexes compared with that of H1N1 WIV alone (Figure S6, Supporting Information), suggesting that the capacity of viral uptake via the transmucosal transport of submucosal DCs was enhanced by CS‐IONzyme. Interestingly, we also found that CS‐IONzyme significantly increased >14% or >16% of direct virus uptake by murine DCs in vitro at 10 or 20 min, respectively, compared to H1N1 WIV alone (Figure S7, Supporting Information). CCL20 and CCL5 that are secreted by mucosal ECs in response to luminal stimuli can attract immature DCs to rapidly migrate into the submucosal regions.^[^ ^6^, ^24^ ^]^ Therefore, individual nasal ECs were isolated from nasal cavity after intranasal immunization of CS‐IONzyme and H1N1 WIV complexes in mice. FCM analysis showed that CS‐IONzyme alone or CS‐IONzyme and H1N1 WIV complexes significantly enhanced the expression of CCL20 but not CCL5 by threefold compared to H1N1 WIV alone (Figure S8, Supporting Information). Observations of cryosections in the nasal tissues by using CLSM showed that DCs were recruited into the CCL20‐enriched epithelial regions (Figure 2a). In addition, the percentages of CCL20‐positive cells in the group of CS‐IONzyme and H1N1 WIV complexes demonstrated a ≈4.4‐fold increase compared to that of H1N1 WIV alone (Figure 2f). Notably, pretreatment with anti‐CCL20 neutralizing antibody, but not with the normal IgG control, significantly attenuated the number of DCs recruited under the mucosa, confirming that CS‐IONzyme‐induced DC recruitment depended on CCL20 secretion (Figure 2g and Figure S9, Supporting Information). In mucosal immune system, antigen‐loaded DCs migrate into draining lymph node through afferent lymphatic, which is critical to initiate the adaptive immune responses.^[^ ^25^ ^]^ In vitro, we found that CS‐IONzyme significantly increased the CCR7 expression, a key migration marker, by ≈6, 5, and 1.9‐fold compared to Medium, CS, and IONzyme, respectively (Figure S10a,b, Supporting Information). Consistently, a chemotaxis assay in transwell chambers on the basis of attraction of mature DCs confirmed that the migration of CS‐IONzyme‐stimulated DCs was remarkably enhanced in response to CCL19, which enriches in the cervical lymph nodes (CLNs), an important draining lymph node of nasal tissues (Figure S10c,d, Supporting Information). For in vivo assay, CLNs were collected to perform FCM analysis 2 h after immunization. We found that CS‐IONzyme remarkably increased the number of H1N1 WIV‐loaded DCs (CD11c^+^ CCR7^+^ H1N1 WIV^+^) that migrated into the CLNs by approximately fourfold, fourfold, or sixfold compared to H1N1 WIV alone, H1N1 WIV plus CS, and H1N1 WIV plus IONzyme, respectively (Figure 2h and Figure S11, Supporting Information). Taken together, these data demonstrated that CS‐IONzyme first strongly stimulated nasal ECs to secrete CCL20, which recruited more DCs into the submucosal regions, and then induced TED formation to capture H1N1 WIV, after which the virus‐loaded DCs quickly migrated into CLNs for initiating the adaptive immune response.

### 2.3 TLR2/4 are Required for CS‐IONzyme‐Induced DC Recruitment

**Figure 3:**
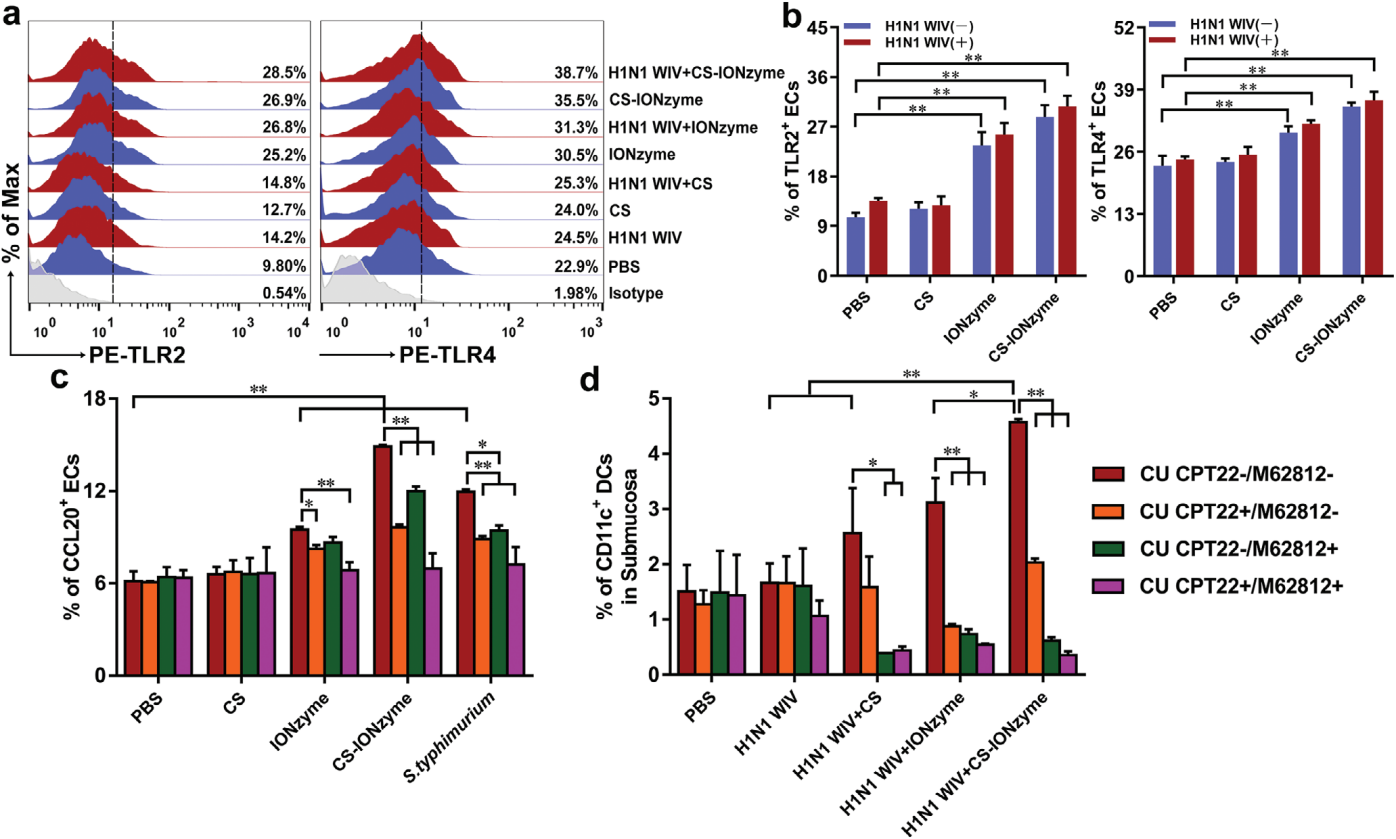
Activation of TLR2 and TLR4 signal pathway by CS‐IONzyme in vivo. a) FCM analysis of TLR2 and TLR4 on the nasal ECs in mice (*n* = 6 per group) after nasally administering of CS‐IONzyme and H1N1 WIV complexes for 0.5 h. The TLR^+^ cells were gated from EPCAM^+^ CD45^−^ cells. b) Quantification of the FCM results as shown in panel (a). c,d) CU CPT22 inhibitor (3 mg kg^−1^) against TLR2, or M62812 inhibitor (20 mg kg^−1^) against TLR4 was administered to mice intraperitoneally 4 h or intravenously 1.5 h respectively before nasally administering of CS‐IONzyme and H1N1 WIV complexes. c) FCM quantitative analysis of CCL20^+^ nasal ECs. The CCL20^+^ ECs were gated from EPCAM^+^ CD45^−^ cells. d) FCM quantitative analysis of submucosal DCs based on the CD11c^+^. Results are from one representative experiment of three performed. Data shown are the means ± s.d. One‐way ANOVA analysis was used to compare the results between different groups. *P < 0.05; **P < 0.01.

To better understand the molecular cues required for DC recruitment and viral capture in the nasal mucosa, we assessed the expression of TLR1–9 on the nasal ECs after intranasal immunization of CS‐IONzyme and H1N1 WIV complexes in mice. FCM analysis showed that IONzyme or CS‐IONzyme did not change the number of the positive ECs expressing TLR1, 3, 5, 6, 7, 8, 9 (Figure S12, Supporting Information). Surprisingly, IONzyme or CS‐IONzyme remarkably increased the number of TLR2^+^ ECs by 2.6 and 2.7‐fold, respectively, compared to PBS group, and TLR4^+^ ECs were increased by 1.3 and 1.6‐fold, respectively (**Figure** **3**a,b). Furthermore, TLR2 or TLR4 inhibitors completely blocked the IONzyme, CS‐IONzyme, or *S. typhimurium*‐induced TLR2 or TLR4 expression on nasal ECs in vivo (Figure S13, Supporting Information).

Of note, the number of CCL20^+^ ECs, and submucosa CD11c^+^ DCs were substantially decreased when using TLR2 or TLR4 inhibitors alone. Moreover, the number of above cells dramatically sunk to the levels of PBS group if TLR2 and TLR4 inhibitors were jointly used (Figure 3c,d; Figures S14 and S15, Supporting Information), suggesting that TLR2 and TLR4 signaling pathways were required for IONzyme or CS‐IONzyme‐induced DC recruitment and viral capture in nasal mucosa.

### 2.4 CS‐IONzyme Promotes DC Maturation via ROS‐Dependent Pathway

**Figure 4:**
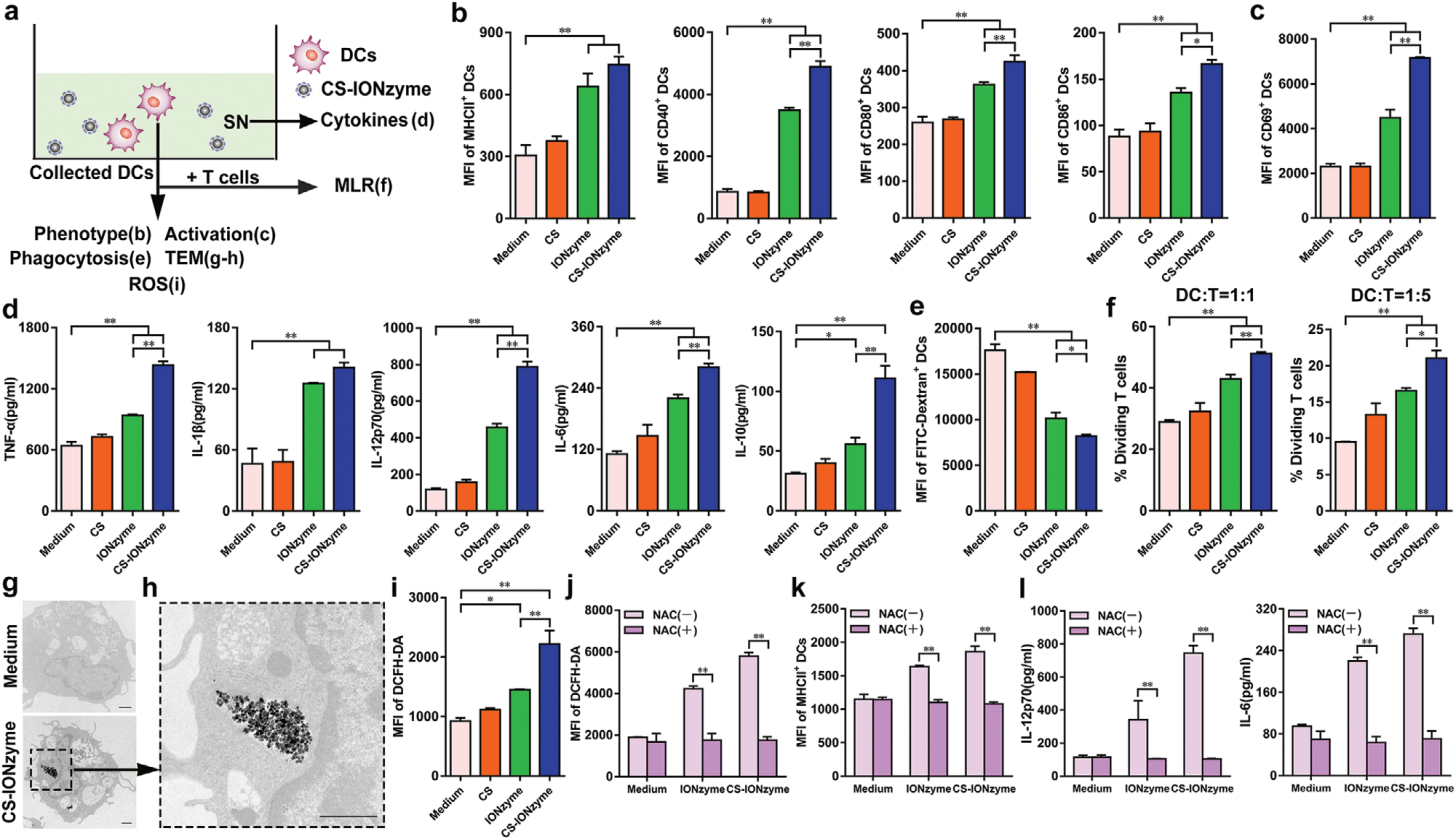
ROS‐mediated DC maturation by CS‐IONzyme in vitro. a) Experimental setting to study DC maturation by induction of CS, IONzyme, and CS‐IONzyme for 24 h. Primary DCs were separated and cultured from murine bone marrow. b) The expressions of phenotypic markers on DCs, including major histocompatibility complex class II (MHCII), CD40, CD80, and CD86, were analyzed by FCM. c) FCM analysis of CD69 expression. d) TNF‐*α*, IL‐1*β*, IL‐12p70, IL‐6, and IL‐10 release in culture supernatants were measured by enzyme‐linked immunosorbent assay (ELISA). e) The endocytosis of FITC‐Dextran by DCs was detected by FCM. f) In the mixed lymphocyte reaction (MLR) experiments, the collected DCs were used in two graded cell numbers (DC/T‐cell ratios: 1:1 and 1:5) to stimulate Carboxyfluorescein Succinimidyl Ester (CFSE)‐labeled allogeneic CD4^+^ T cells (5 × 10^5^ responder cells per well). After 5 days, proliferation was detected by FCM. g,h) TEM image of internalization of CS‐IONzyme in the lysosome of DCs. An enlargement of the region in the black frame in panel g (scale bar: 1 µm) is show in panel h (scale bar: 1 µm). i) Cells were stained with DCFH‐DA and analyzed by FCM for ROS detection. j–l) DCs were pretreated with ROS scavengers (NAC, 3 mm) or not for 1 h, ROS, MHCII, and cytokine secretion (IL‐12p70 and IL‐6) were detected. All of the data are presented as means ± s.d. of three replicates and are representative of three independent experiments. Statistical significance is assessed by One‐way ANOVA analysis to compare the results between the different groups (b–f) or by unpaired Student's two‐sided *t*‐test to compare between the two groups (j–l). *P < 0.05; **P < 0.01. SN: supernatant.

It is a critical step that DCs transfer from immature state to mature state for the subsequent antigen‐specific immune response.^[^ ^26^ ^]^ Therefore, DCs were incubated with CS, IONzyme, and CS‐IONzyme for 24 h in vitro, respectively, then the DC maturation was assessed (**Figure** **4**a). As shown in Figure 4b and Figure S16a–d (Supporting Information), the expressions of MHCII, CD40, CD80, and CD86 were remarkably upregulated in the group of IONzyme/CS‐IONzyme compared with that of control. Of note, CS‐IONzyme significantly increased the expression of above phenotype markers in comparison to IONzyme, while CS alone did not change their expression. Next, we also found that CS‐IONzyme enhanced the expression of activation marker CD69 by 3.5‐fold compared to the control (Figure 4c and Figure S16e, Supporting Information). Furthermore, we assessed the functional maturation of DCs by detecting the release of cytokines, including TNF‐*α*, Interleukin (IL)‐1*β*, IL‐12p70, IL‐6, and IL‐10 from the supernatants. As expected, DCs responded to IONzyme/CS‐IONzyme with significant increases in these cytokine secretions compared with control (Figure 4d). The competence for endocytosis in immature DCs is efficient but lost during the maturation stage.^[^ ^27^ ^]^ As shown in Figure 4e and Figure S16f, Supporting Information, IONzyme/CS‐IONzyme decreased the endocytosis capability of DCs by ≈1.8 and 2.3‐fold compared to the control, respectively, suggesting that DCs have been converted into mature stage. To estimate whether DCs might be as fully functional antigen‐presenting cells, the treated DCs were evaluated for their ability of stimulating allogeneic CD4^+^ T cells. Similarly, DCs that were from the group of IONzyme/CS‐IONzyme obviously promoted the proliferation of allogeneic CD4^+^T lymphocytes compared with that from control (Figure 4f and Figure S16g, Supporting Information). We also found that IONzyme/CS‐IONzyme can directly stimulate the proliferation of splenic lymphocytes, implying that it can be developed a potential adjuvant (Figure S17a, Supporting Information). Collectively, these findings indicated that IONzyme/CS‐IONzyme had the capability of enhancing the maturation of DCs. Furthermore, we observed that IONzyme/CS‐IONzyme were located within the lysosome of DCs by using TEM (Figure S18, Supporting Information, Figure 4g,h). CLSM images confirmed that CS‐IONzyme co‐localized with lysosomes (Figure S19, Supporting Information), the highly acidic microenvironment of which favors IONzyme with POD‐like activity, which can induce ROS production.^[^ ^19^ ^]^ We found that a marked increase in DCFH‐DA staining was detected with CS‐IONzyme, which was up to 2.5‐fold compared to levels measured following treatment with medium (Figure 4i and Figure S20, Supporting Information). Furthermore, we observed that a strong ROS generation by CS‐IONzyme was occurred in lysosomes (Figure S21, Supporting Information). These data indicated that a ROS burst was initiated by adding CS‐IONzyme to DCs. Consistently, we found that IONzyme/CS‐IONzyme can directly induce ROS generation in splenic lymphocytes (Figure S17b, Supporting Information). To understand the correlation between the DC maturation and ROS, we next used N‐Acetyl‐L‐cysteine (NAC) as ROS scavengers (Figure 4j and Figure S22a, Supporting Information). After blocking the intracellular ROS production, the phenotype marker expressions (MHCII and CD40) (Figure 4k and Figure S22b‐d, Supporting Information), and cytokine secretions (IL‐12p70, IL‐6, TNF‐*α*, IL‐1*β*, and IL‐10) (Figure 4l and Figure S22e, Supporting Information) of DCs were suppressed completely, suggesting that IONzyme/CS‐IONzyme‐induced DC maturation depended on ROS pathway.

### 2.5 CS‐IONzyme‐Based Mucosal Vaccination Offers a Strong Adaptive Immunity

**Figure 5:**
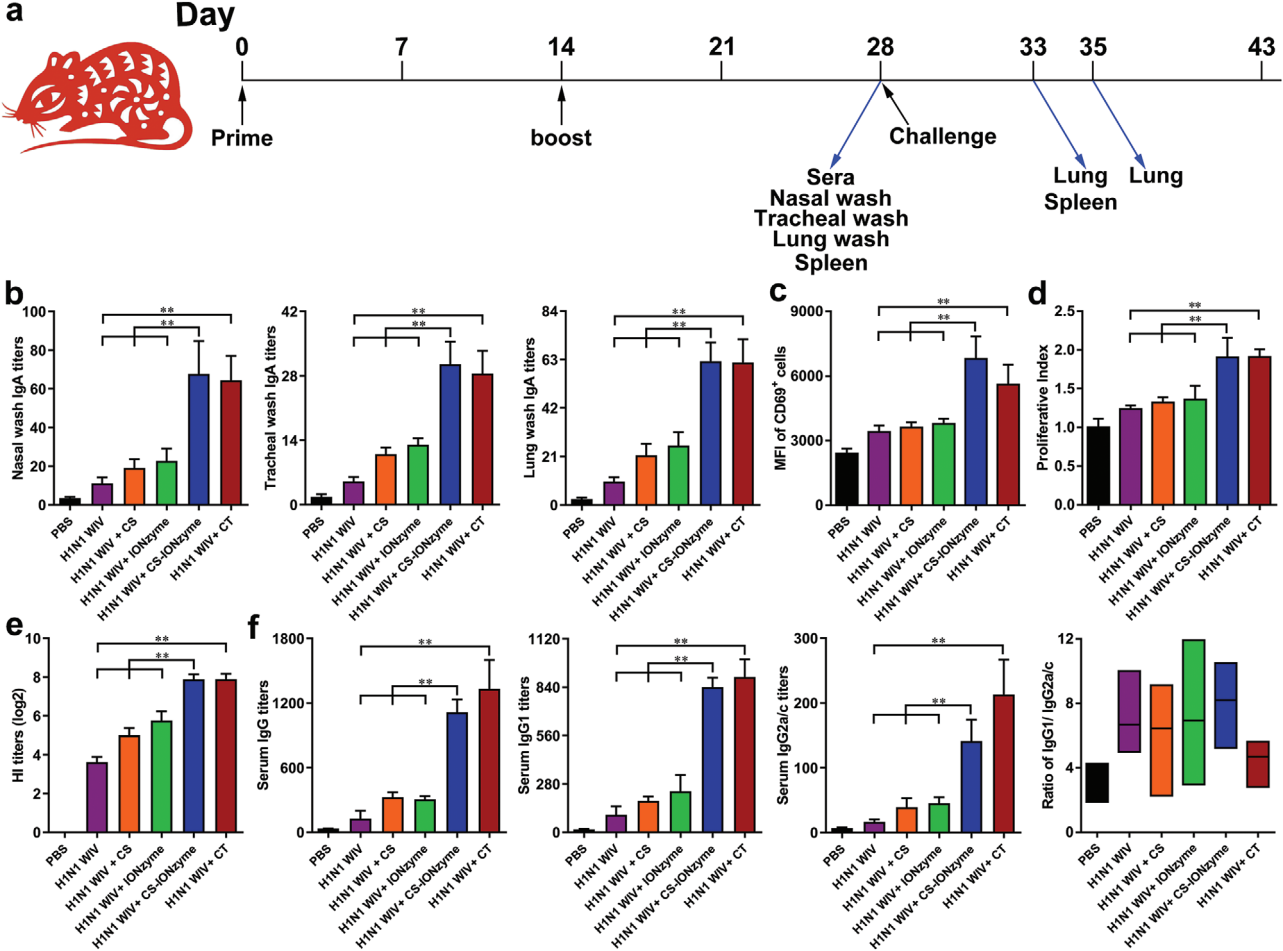
The induction of mucosal and systemic immune responses by CS‐IONzyme‐based influenza vaccine after intranasal immunization in mice. a) Scheme of immunization (*n* = 10 per group), challenge, and sampling. b) At 28 days after the primary immunization, H1N1 WIV‐specific mucosal IgA titers in nasal wash, tracheal wash, and lung wash were determined by endpoint ELISA. c,d) Splenocytes were restimulated by H1N1 WIV (10 µg mL^−1^) following 72 h in vitro. c) FCM analysis of splenocyte activation (assessed as CD69 expression). d) CCK‐8 assay was used to analyze splenocyte proliferation. e) Serum hemagglutination inhibition (HI) levels were detected by HI assay using 4 hemagglutinating units of the influenza virus strain. f) H1N1 WIV‐specific IgG, IgG1, and IgG2a/c titers in serum were determined by endpoint ELISA, and the radio of between IgG1 and IgG2a/c for Th1/Th2 skew was also analyzed. Results are from one representative experiment of two performed. All of the data are presented as means ± s.d. Statistical significance is assessed by One‐way ANOVA analysis to compare the results between different groups. *P < 0.05; **P < 0.01.

To verify whether CS‐IONzyme might be an effective component in response to H1N1 WIV, the mice were intranasally immunized two times with H1N1 WIV alone, H1N1 WIV plus CS, H1N1 WIV plus IONzyme, H1N1 WIV and CS‐IONzyme complexes, or H1N1 WIV in combination with a known mucosal adjuvant CT (**Figure** **5**a). The secretory IgA (s‐IgA) antibody located on the surface of mucosa, which is critical for antigen‐specific mucosal protection, was detected. As shown in Figure 5b, in nasal wash samples, H1N1 WIV and CS‐IONzyme complexes or H1N1 WIV plus CT was found to induce much higher IgA specific antibody titers by 6.3‐fold and sixfold compared to H1N1 WIV alone. Similar trends of IgA levels were also found in tracheal and lung wash samples, suggesting a wonderful efficacy of CS‐IONzyme on mucosal immune responses in the lower respiratory tract. Moreover, splenic lymphocytes were isolated from immunized mice, and restimulated with H1N1 WIV in vitro. We found that the CD69 expression (Figure 5c and Figure S23, Supporting Information), the proliferative index (Figure 5d), and the percentage of CD3^+^CD4^+^splenic T cells were all markedly increased in the group of H1N1 WIV and CS‐IONzyme complexes compared with that of the antigen alone (Figure S24, Supporting Information). Besides, the serum was collected from the immunized mice at 28 days and the hemagglutination‐inhibiting (HI) antibody was detected. As shown in Figure 5e, the HI titers from the group of H1N1 WIV and CS‐IONzyme complexes showed a significant increase by ≈4log2 compared to H1N1 WIV group, and the results were similar with that of H1N1 WIV plus CT group. Furthermore, we also found that serum antigen‐specific IgG, IgG1, and IgG2a/c antibody titers induced by H1N1WIV and CS‐IONzyme complexes were substantially greater than antigen‐alone immunization by 8.9, 8.4, and 8.5‐fold, respectively (Figure 5f). Additionally, IgG1/IgG2a ratio was >1 in the group of H1N1WIV alone or H1N1 WIV and CS‐IONzyme complexes, indicating that H1N1 WIV alone or together with CS‐IONzyme induced a primarily Th2‐type antibody response. Altogether, these results demonstrated that intranasal vaccination with CS‐IONzyme‐based influenza vaccine strongly induced mucosal and systemic immune responses in mice.

### 2.6 CS‐IONzyme‐Based Mucosal Vaccination Offers A Strong Influenza Protection

**Figure 6:**
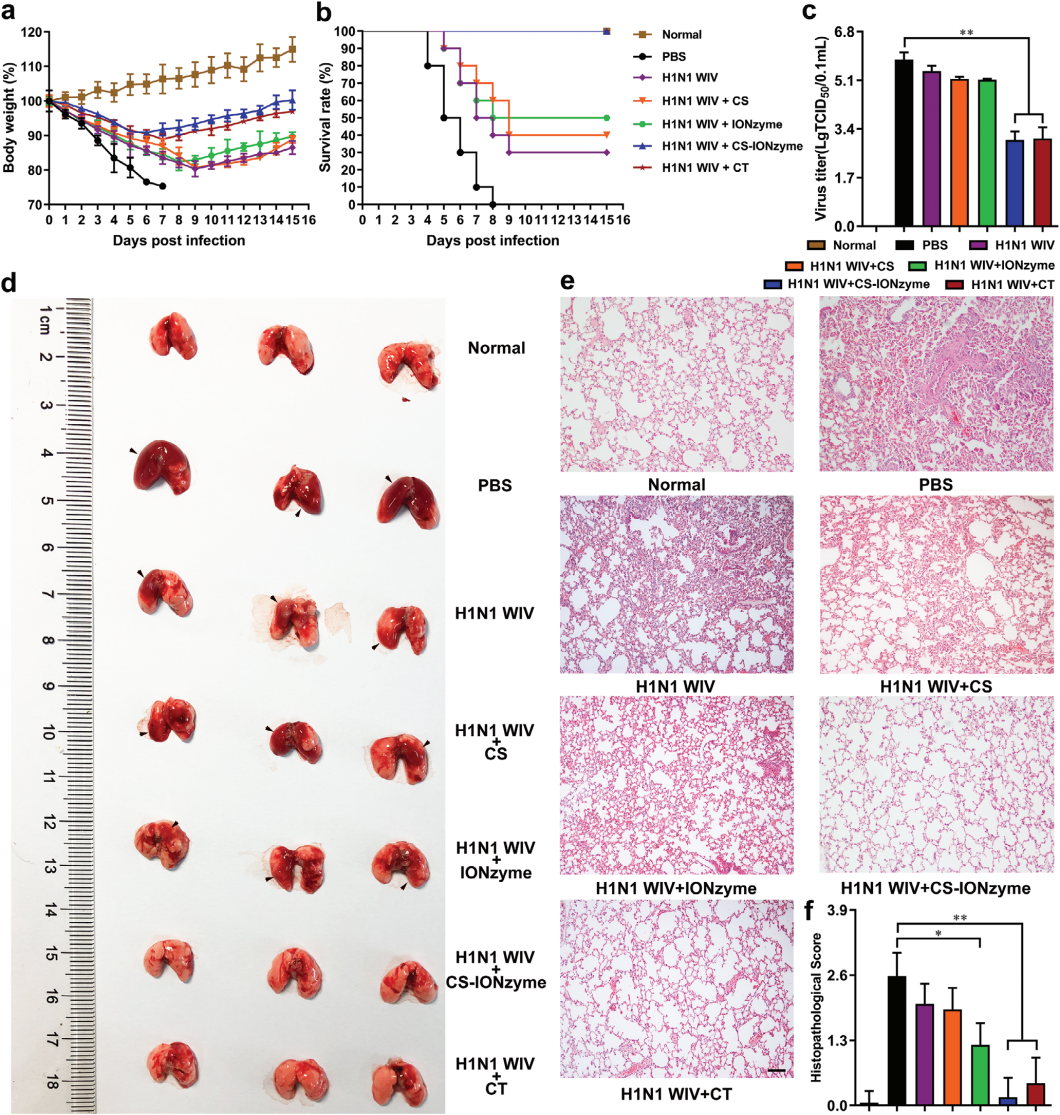
Intranasal immunization with CS‐IONzyme‐based influenza vaccine protects mice against H1N1 influenza virus infection. The vaccinated mice were challenged with 10^6^ EID_50_ of H1N1 influenza virus 28 days post primary immunization. a) Body weight changes. b) Survival rates. Mice who lost more than 25% of their body weight were euthanized according to guidelines. *n* = 10 mice per group. c) Virus load in the lung (*n* = 5 per group) on day 5 p. i. d) The pathological changes in lungs (*n* = 3 per group) on day 7 p. i. e) Representative histopathological changes in H&E‐stained lung tissues (*n* = 3) on day 7 p. i. Bars: 500 µm. f) Histopathologic scores in the lungs. Data shown represent the means ± s.d. Results are from one representative experiment of two performed. Statistical significance is assessed by One‐way ANOVA analysis to compare the results between different groups. *P < 0.05; **P < 0.01.

To evaluate the protective efficacy of CS‐IONzyme‐based mucosa delivery vaccine, intranasally immunized mice were challenge with 10^6^ 50% egg infectious dose (EID_50_) of H1N1 (A/PR/8) virus 28 days post primary immunization. The changes in body weight and survival rates were monitored daily for 2 weeks post‐challenge. As shown in **Figure** **6**a,b, mice immunized with H1N1 WIV and CS‐IONzyme complexes or H1N1 WIV plus CT recovered after a slight loss of body weight and all the mice in that group survived to the end of the experimental period, demonstrating a 100% protective efficacy. The virus titer in the lung from the group of H1N1 WIV and CS‐IONzyme complexes was significantly lower than that from the H1N1 WIV group by 1.9‐fold (Figure 6c). However, the challenged mice from the group of H1N1 WIV alone showed a severe weight loss, part death with a 30% protection, and high virus load in the lung. On day 5 and 7 post infection (p. i.), the challenged mice (PBS group) showed a series of severe pathological changes in lungs, including swell, hyperemia, and hemorrhage. Meanwhile, we found that the lung tissues exhibited severe pneumonia with inflammatory cellular infiltration, alveolar wall edema and thickening, and pneumonorrhagia by using an H&E staining method. Additionally, severe lesions were found in the enlargement of the spleens on day 5 p. i. However, H1N1 WIV and CS‐IONzyme complexes‐immunized mice showed a slight pathologic and histopathological changes (Figure 6d–f; Figures S25 and S26, Supporting Information). These data demonstrated that intranasal vaccination with CS‐IONzyme‐based H1N1 inactivated vaccine protected mice from a lethal influenza challenge.

## 3 Discussion

Our work designed a novel mucosal vaccine against influenza virus basing on CS‐IONzyme. Strikingly, CS‐IONzyme‐H1N1 WIV complex vaccine provide a 100% protective immunity against influenza virus with a lethal attack, after intranasal immunization by two times in mice. As a potential mucosal adjuvant alternative, CS‐IONzyme, as a catalytic adjuvant, demonstrates the following features: i) CS‐IONzyme poses high biosecurity, high biostability, high dispersibility in aqueous solution basing on the blockade of aggregation by CS, low cost, and easy preparation. ii) CS‐IONzyme possesses a powerful adhesion ability for antigen mucosal delivery against mucociliary clearance. iii) CS‐IONzyme can strongly catalyze mucosal immune system through enhancing the innate immune response of nasal ECs and submucosal DCs via TLR2/4 pathway and ROS pathway, respectively. iv) CS‐IONzyme facilitates H1N1 WIV to strongly trigger the mucosal and systemic adaptive immune responses. These features together provide potential advantages for practical applications of CS‐IONzyme in the prevention and control of influenza.

Importantly, in the design of mucosal vaccine, how to effectively deliver the antigens to cross the mucosal barriers is a key step for initiating subsequent antigen‐specific immune response. Unlike intramuscular immunization, nasal antigens must cross various barriers (epithelium in compact arrangement by expressed tight junction proteins, mucociliary clearance, and mucus) before they contact with submucosal DCs, as professional antigen‐presenting cells, which play a central role in the mucosal immune system.^[^ ^6^, ^28^ ^]^ In particular, the viral replication in the mucosa is completely abolished for inactivated influenza vaccine, resulting in an immunity failure, because dead viruses are easy to be cleared by cilia and mucus. Another important reason is that both WIV particles and nasal mucosa pose negative charges, resulting in that virions are hardly to adhere onto the surface of mucosa. Therefore, we introduced lower dose CS onto the IONzyme and converted it to the state of positive charges. When CS‐IONzyme and H1N1 WIV formed a complex, they still posed positive charges, which remarkably improved the adhesion amount and residence time of H1N1 WIV onto the nasal mucosa. Additionally, how to assist adhered antigens to effectively cross the mucosa and be captured by submucosal DCs, which is also a difficulty. A seminal study by Rescigno et al. has demonstrated that submucosal DCs can sense luminal “danger signals” and fast form TEDs to penetrate intestinal epithelial monolayers to sample bacteria without destroy intercellular tight junction proteins,^[^ ^29^ ^]^ implying that a novel antigen delivery strategy that would be developed through mobilizing broad submucosal DCs to capture luminal antigens using their TEDs. As expected, we found that DCs were strongly recruited into the nasal mucosa and sent their TEDs to capture the luminal H1N1 WIVs in the induction of CS‐IONzyme, indicating that CS‐IONzyme might be a strong stimulant as a “foreign danger” to swindle DC recruitment and TED formation. Additionally, CS might partly open the tight junctions between ECs,^[^ ^30^ ^]^ which help DCs easily form TEDs. We further confirmed that the chemokine CCL20 rather than CCL5 secreted from nasal ECs played a key role on attracting DCs into the submucosal regions to send their TEDs for viral uptake. We then asked how CS‐IONzyme can induce nasal ECs to produce abundant CCL20. As known, toll‐like receptors (TLRs), as pattern recognition receptors (PRRs), recognize danger signals referred to as pathogen‐associated molecular patterns (PAMPs) to activate both innate immunity and instruct the development of antigen‐specific acquired immunity.^[^ ^31^ ^]^ Previously, lipopolysaccharide, flagellin, and CpGs can be recognized by TLR4, TLR5, and TLR9, respectively, which can enhance DC recruitment and TED formation.^[^ ^6^, ^32^ ^]^ Surprisingly, we provided evidence that IONzyme, as an inorganic substance, can strongly activate TLR2/4 signals, but not TLR1, 3, 5, 6, 7, 8, or 9, which could be speculated a possible direct receptor‐ligand interaction between TLR2/4 and IONzyme. However, we did not observe the TLR2/4 expression can be promoted when the interaction between IONzyme and DCs in vitro (Figure S27, Supporting Information), implying that IONzyme might indirectly activate TLR2/4 signals. In nasal situ, it cannot be ignored that an amount of mucus exists the surface of the mucosa, which is a complex aqueous mixture of glycoprotein, lipid, salts and cellular debris.^[^ ^33^ ^]^ Specially, the airway secretions also contain a mixture of lipid components including free fatty acids, triglycerides, cholesterol and phospholipids.^[^ ^34^ ^]^ Interestingly, oxidized phospholipids can induce TLR2‐ or TLR4‐dependent immune responses.^[^ ^35^ ^]^ Previously, Wang et al. has reported that Fe_3_O_4_ nanoparticles induced lipid peroxidation by POD‐like activity, which requires acidic pH.^[^ ^36^ ^]^ Therefore, basing on the environment of acidic mucus (pH 5.5–6.5),^[^ ^37^ ^]^ we deduced that IONzyme with a POD‐like activity might induce lipid peroxidation of phospholipids existing within the nasal mucus, and then oxidized products activate TLR2‐ and/or TLR4‐ signaling pathway, which further induce CCL20 secretion to enhance the DC recruitment and TED formation for antigen uptake. In this process, CS doping increased the effect of IONzyme because of the improvement of the POD‐like catalytic capacity.

In the mucosal immune system, DCs are first mobilized into the submucosa, and efficiently capture, internalize and deliver luminal antigens into acidic lysosomal compartments for antigen processing.^[^ ^20^ ^]^ After then, they need to rapidly mature and migrate into draining lymph node to present antigen to T lymphocytes for initiating antigen‐specific immune responses. Therefore, how to deliver antigens into lysosomes and quickly improve the DC maturation is a key step for vaccine design. Previously, IONzyme demonstrated dual enzyme‐like activities, peroxidase and catalase, under acidic and neutral pH, respectively, which can regulate intracellular ROS levels.^[^ ^11^ ^]^ For instance, antitumor^[^ ^17^ ^]^ and anti‐bacterial^[^ ^18^ ^]^ application of IONzyme were mainly based on their POD‐like activity, which can induce ROS generation. However, catalase activity have the ability to scavenge ROS, which can guide it as an antioxidant.^[^ ^38^ ^]^ In our experiment, we found that a large amount of IONzyme were effectively captured by DCs and delivered into lysosomes by using TEM and CLSM, the highly acidic microenvironment of which would favor the production of POD‐like activities. As expected, the cell assays further showed that IONzyme resulted in higher intracellular ROS levels. ROS generation may be as a potential strategy for moderating DC maturation.^[^ ^22^ ^]^ After blocking the ROS pathway by NAC inhibitor, we found that DCs were hardly turned into mature state. These results suggested that IONzyme posed a typical adjuvant feature through its POD‐like activity to boost ROS generation for DC maturation. In addition, CS doping in IONzyme significantly improved the catalytic efficiency for enhancing the ROS generation in lysosomes and subsequent DC maturation, implying that CS‐IONzyme was likely to be a promising adjuvant candidate for vaccine application.

Accompanied by DC maturation, CS‐IONzyme also enhanced the migration of H1N1 WIV‐loaded DCs with CCR7 upregulation into the draining CLNs to present the antigens to the T lymphocytes, which play a critical role in initiating the adaptive immune response. As expected, CS‐IONzyme facilitated H1N1 WIV to strongly trigger antigen‐specific mucosal and systematic immune response. S‐IgA antibodies are the predominant secretory immunoglobulin that mediate adaptive humoral immune defenses at mucosal surfaces,^[^ ^39^ ^]^ which can effectively establish a “frontline immunity” at the invasion site of influenza virus, thus avoiding the manifestation and dissemination of an infection.^[^ ^5b^ ^]^ IgA also provides some cross‐protection against drift and shift virus variants.^[^ ^3b^ ^]^ In addition, according to the “Common Mucosal Immune System” theory, IgA also can be induced at other mucosal sites, including tracheal, lung, and gastrointestinal tract.^[^ ^40^ ^]^ Mucosal immunization also evokes systemic antibody IgG production. However, conventional systemic intramuscular influenza vaccines only induce serum IgG, which is difficult to reach the mucosa site for virus neutralization. In addition, our vaccine also induced Th2‐type antibody responses and resulted in a strong and rapid induction of antibody titers, which makes them potentially suitable for the pandemic situation where likely only one vaccination can be achieved before the first wave of the pandemic.^[^ ^41^ ^]^ Basing on multiple advantages of mucosal immunization, CS‐IONzyme‐based mucosa delivery vaccine provided a strong protective immunity against influenza virus with a lethal attack.

## 4 Conclusion

In conclusion, we have demonstrated that a novel CS‐IONzyme‐based influenza vaccine with dual targeting both nasal mucosa and submucosal DCs, leading to a robust immune response against influenza virus. Utilizing an excellent POD‐like activity of CS‐IONzyme, a novel catalytic immune‐adjuvant against the bottleneck of inactivated virus mucosal vaccines is successfully developed by both fully mobilizing broad submucosal DCs to form TEDs for uptake of luminal antigens and strongly activating DC maturation. Our research expanded the biomedical applications of IONzyme basing on their enzyme catalytic property. We conclude that CS‐IONzyme has a potential to be used in a wide variety of needle‐free, intranasal‐administered, and powerful‐protective mucosal vaccine against H1N1, H5N6, H7N9, and other influenza virus.

## 5 Experimental Section

##### Ethics Statement

The Jiangsu Administrative Committee for Laboratory Animals (Permission number: SYXKSU‐2007‐0005) approved all of the animal studies according to the guidelines of Jiangsu Laboratory Animal Welfare and Ethical of Jiangsu Administrative Committee of Laboratory Animals. All experiments involving live viruses and animals were performed in the authorized animal biosafety level 2 (ABSL‐2) facilities at Yangzhou University.

##### Materials

NaOAc was from Sinopharm Chemical reagent. FeCl_3_, ethylene glycol, CT, chitosan (CS), and N‐Acetyl‐L‐cysteine were from Sigma‐Aldrich. CU CPT22 (TLR1/2 inhibitor) was from Absin. M62812 (TLR‐4 inhibitor) was from Tocris. Fluorescein isothiocyanate (FITC) was from Aladdin. Recombinant mouse GM‐CSF, IL‐4, and CCL19 were from PeproTech. Lyso‐Tracker Red and CCK‐8 kit were from Beyotime. Fixable viability stain 620 (FVS620) and Fixable viability stain 780 (FVS780) were from BD PharMingen. Antibodies used for flow cytometry and immunofluorescence were listed in Tables S1 and S2, Supporting Information, respectively. Horseradish peroxidase‐conjugated anti‐mouse IgG, IgG1, and IgG2a/c were from Santa Cruz. Horseradish peroxidase‐conjugated anti‐mouse IgA was from Southern Biotech.

##### Synthesis and Characterization of CS‐IONzyme

The solvothermal method was used to synthesize CS‐IONzyme according to previous literature.^[^ ^42^ ^]^ Briefly, 0.82 g FeCl_3_ was dissolved in 40 mL ethylene glycol to form a clear solution. Next, 3.6 g NaOAc and 0.1 g CS were added with vigorous stirring for 30 min. The mixture was transferred to a 50 mL Teflon‐lined stainless‐steel autoclave and reacted at 200 °C for 12 h. After the autoclave cooled to room temperature, the black precipitate was collected and washed three times with ethanol, and then dried at 60 °C for 3 h. The synthesized nanoparticles were determined with SEM (Hitachi S‐4800), TEM (JEOL JEM‐1400), X‐ray photoelectron spectroscopy (XPS, Thermo Scientific ARL Quant’ X), thermal gravimetric analyzer (TGA, PerkinElmer Pyris 1 TGA), and nanoparticle zeta potential and size analyzer (Malvern Instrument Nano‐ES90). The peroxidase‐like activity of CS‐IONzyme was measured by monitoring the absorbance change at 652 nm on a microplate reader (Tecan, Switzerland). The kinetic assays were performed using 10 µg of CS‐IONzyme in 200 µL of reaction buffer (0.1 m NaOAc buffer, pH 4.5) in the presence of H_2_O_2_ and TMB. We performed the kinetic analysis of CS‐IONzyme by varying the concentrations of H_2_O_2_ with 0.8 mm TMB. All reactions were monitored by measuring the absorbance (652 nm) at different reaction times, and the Michaelis–Menten constant was calculated according to the Michaelis‐Menten saturation curve as described previously.^[^ ^42^ ^]^ The bio‐stability was evaluated according to a previous method,^[^ ^43^ ^]^ CS‐IONzyme (5 mg mL^−1^) were tested in simulated body fluid (SBF) and cell culture medium (EMEM, Sigma) completed with 10% of fetal bovine serum. The samples were maintained under continuous stirring (150 rpm) at 37 °C. At selected times (0 h, 1 h, 24 h, 72 h, 7 d, and 14 d), the samples were collected to perform the morphologic observation by TEM/SEM and detection of POD‐like activity.

##### Preparation of H1N1 WIV and CS‐IONzyme Complexes

H1N1 influenza virus (A/Puerto Rico/8/34) were identified and stored by our laboratory, and then purified by using a discontinuous sucrose density gradient centrifugation according to a previously described method.^[^ ^6b^ ^]^ Inactivated viruses were prepared at 56 °C for 0.5 h and tested for complete loss of infectivity by inoculation into 10‐day‐old specific‐pathogen‐free (SPF) embryonated eggs for three passages. The quantity of viruses was measured by BCA protein assay kit (Thermo Fisher). The HA protein concentration was about 35% of the total protein, as determined previously.^[^ ^44^ ^]^ H1N1 WIV and CS‐IONzyme were dissolved in phosphate‐buffered saline (PBS, 0.01M) to adjust the concentration to 1.5 and 10 mg mL^−1^, respectively. H1N1 WIV and CS‐IONzyme complexes were prepared by adding H1N1 WIV solution into CS‐IONzyme solution at equal volume and vortexed for 0.5 h at room temperature. H1N1 WIV and CS‐IONzyme complexes were analyzed using TEM. For viral labeling, viruses were labeled with the NHS (N‐hydroxysuccinimide) ester fluorescent probe DyLight 488 or 550 (Thermo Fisher) according to the instructions provided by the manufacturer. Unincorporated dye was removed using commercial fluorescent dye removal columns (Thermo Fisher).^[^ ^44^ ^]^ For CS‐IONzyme labeling, CS‐IONzyme and FITC were dissolved in acetic acid and DMSO to adjust the concentration to 10 mg mL^−1^, respectively. CS‐IONzyme solution and FITC solution were mixed (v/v, 1:5) and vortexed for 4 h at room temperature, washed with 70% ethanol, and then dried in vacuo for 4 h.

##### Immunogenicity Study

6‐week‐old C57BL/6 mice were divided into six groups (*n* = 10 per group), and immunized intranasally at 0 and 14 days with 20 µL PBS, or H1N1 WIV (containing 5 µg HA) alone, or H1N1 WIV (containing 5 µg HA) plus CS (8.5 µg), or H1N1 WIV (containing 5 µg HA) plus IONzyme (100 µg), or H1N1 WIV (containing 5 µg HA) and CS‐IONzyme (100 µg) complexes, or H1N1 WIV (containing 5 µg HA) plus CT (2 µg), respectively. The mice were euthanized and samples were collected 28 days after the primary immunization. Blood was collected to separate sera. Nasal, tracheal and lung lavage fluid were obtained by washing the organs with 0.5, 0.2, and 0.5 mL sterile PBS, respectively. HI test for sera antibodies against the H1N1 strain was carried out as previously described.^[^ ^45^ ^]^ Antigen‐specific mucosal wash secretory IgA antibodies and serum antibodies (total IgG, IgG1, and IgG2a/c) were determined by ELISA using horseradish peroxidase‐conjugated anti‐mouse IgA, total IgG, IgG1, and IgG2a/c as described previously.^[^ ^6b^ ^]^ Here, an anti‐IgG2a isotype which cross‐reacts with IgG2c was used and titers were reported as IgG2a/c titers.^[^ ^46^ ^]^ In addition, splenocytes were isolated from the immunized mice and then restimulated with H1N1 WIV (10 µg mL^−1^) following 72 h in vitro. CD69 activation was performed by FCM and proliferative response was measured using the CCK‐8 assay.

##### Virus Challenge

28 days post primary immunization, vaccinated mice were anesthetized and then inoculated intranasally challenged with 10^6^ EID_50_ of H1N1 influenza virus. Body weight changes and survival rates of infected mice (*n* = 10 per group) were monitored for 15 days as described previously.^[^ ^15^ ^]^ Lung tissues (*n* = 3 per group) were collected for pathological or histopathological examination, or virus titration (*n* = 5 per group) at 5 or 7 days post infection (p.i.). Microscopic lesions were evaluated by two blinded veterinary pathologists. Scoring of lesions was based on scales adapted from the work of Gauger et al.^[^ ^47^ ^]^ For detection of viral titers, each tissue sample was homogenized in 0.4 mL of PBS containing antibiotics and centrifuged at 8000 rpm for 10 min, and 0.1 mL of supernatant was used to inoculate confluent Madin–Darby canine kidney cell monolayers by using 96‐well plates under 37 °C for 1 h from initial dilutions of 1:10. Afterward, the supernatants were replaced with medium (1% fetal bovine serum (FBS) in Dulbecco's modified Eagle medium) and incubated at 37 °C for 72 h. The presence of the virus in the supernatant was assayed by measuring the hemagglutinating activity.^[^ ^15^ ^]^ The TCID_50_ per 0.1 mL was calculated using the Reed–Muench method as described previously.^[^ ^48^ ^]^

##### Tissue Collection and Cryosection

Mice were nasally administered DyLight 488 or 550 labeled H1N1 WIV (5 µg HA) alone or combination with CS (8.5 µg), IONzyme (100 µg), CS‐IONzyme (100 µg), or the same volume of PBS. Then the mice were anesthetized and sacrificed after indicated time of inoculation. After removing the excess tissues, the noses were fixed in 4% paraformaldehyde overnight. Next, the noses were decalcified for 7 days in decalcifying fluid (10% EDTA, PH 7.4) and then frozen in embedding medium (OCT, Torrance, CA). Cryosections (8 µm) were cut and processed for immunofluorescence as below. For FCM analysis of nasal ECs,^[^ ^6b^ ^]^ the murine nasal tissues were separated without additional skins, muscles, and CLNs. Subsequently, the nasal‐associated lymphoid tissues (NALTs) located on the basilar nasal tissues were removed, and the individual cells were isolated by Type XIV protease (0.5%), Type IV collagenase (0.1%), and hyaluronidase (0.1%) at 37 °C for 0.5 h, and then were through a 100‐µm cell strainer. Nasal ECs were gated from EPCAM^+^CD45^−^ cells according the previous study.^[^ ^49^ ^]^

##### Immunofluorescence and Confocal Microscopy

Briefly, the cryosections were permeabilized in 0.2% Triton X‐100 in PBS for 5 min, and then blocked with 5% bovine serum albumin for 1 h followed by incubation with primary antibodies overnight at 4 °C. The bound antibodies were labeled with different fluorescent secondary antibodies for 1 h at room temperature. The DCs were immunolabeled with Armenian hamster anti‐CD11c mAb followed by Alexa Fluor 649‐conjugated goat anti‐Armenian hamster IgG. CCL20 was labeled with rabbit anti‐CCL20 polyclonal antibody followed by Alexa Fluor 488‐ conjugated goat anti‐rabbit IgG. As a control, the specificity of the antibodies and the labeling procedure were tested with the isotype control antibodies. The cryosections were observed by a Leica TCS SP8 STED confocal microscope (Leica Microsystems, Wetzlar, Germany). Serial sections were collected by Z projection with 0.5 µm increments on the *z* axis. 3D images were rendered using Imaris 7.2 software.

*Generation of DCs*: DCs were isolated and cultured using our advanced method.^[^ ^6b^ ^]^ In brief, bone marrow was obtained from the tibias and femurs of C57BL/6 mice and cultured in complete medium (RPMI‐1640 (Invitrogen) with 10% FBS, 1% penicillin/streptomycin, 10 ng mL^−1^ GM‐CSF and IL‐4). After 60 h of culture, medium was gently discarded and fresh medium was added. On day 6, nonadherent and loosely adherent cells were harvested and centrifuged to remove debris and dead cells, then cultured overnight. Only cultures with >90% cells expressing CD11c by FCM were used.

##### Phenotype, Migration, and Cytokine Assay

In vitro, the DCs were incubated with CS (8.5 µg mL^−1^), IONzyme (100 µg mL^−1^), or CS‐IONzyme (100 µg mL^−1^) for 24 h. Next, the DCs were harvested and incubated with FITC‐MHCII, PE‐CD40, PE‐CD80, FITC‐CD86, PE‐CCR7, or the respective isotypes at 4 °C for 30 min as per the manufacturer's guidelines. After being washed three times with PBS, the cells were analyzed by FCM. The levels of TNF‐*α*, IL‐1*β*, IL‐12p70, IL‐6, and IL‐10 in the culture supernatants were measured by using ELISA kits (eBioscience, San Diego, CA) and performed according to manufacturer's instructions.

##### Allogeneic Mixed Lymphocyte Reaction Assay

Responder T cells were purified from BALB/c splenic lymphocytes by using a CD4^+^ T cell isolation kit (Miltenyi Biotech, Germany) and labeled with carboxyfluorescein succinimidylester (CFSE) (Invitrogen, Carlsbad, CA) according to manufacturer's instructions. Then these cells were co‐cultured in duplicate with DCs (DC/T cell ratios of 1:1 and 1:5) for 5 days and detected by FCM.

##### ROS Generation Assay

In brief, after being treated with CS (8.5 µg mL^−1^), IONzyme (100 µg mL^−1^), or CS‐IONzyme (100 µg mL^−1^) for 24 h, DCs were collected and incubated with 10 µm DCFH‐DA (Beyotime) at 37 °C for 20 min. After washing three times with PBS, DCs were analyzed by FCM. To detect lysosomal ROS,^[^ ^50^ ^]^ the treated DCs were incubated with 2 µm RedoxSensor Red CC‐1 and 2 µm LysoSensor Green DND‐189 as a lysosomal marker (both Thermo) at 37 °C for 30 min in the dark, and then DCs were observed by using confocal microscopy.

##### Statistical Analysis

Results were expressed as the means ± s.d. and analyzed with GraphPad Prism 8 software (San Diego, CA). Unpaired Student's two‐sided *t*‐test was employed to determine the differences between the two groups. To compare multiple groups, One‐way ANOVA with Tukey's post hoc test was performed by using SPSS 17.0. *P < 0.05, **P < 0.01.

## Conflict of Interest

The authors declare no conflict of interest.

## Supporting information

Supporting Information
