# Supplementary material for: Mucosal Vaccination for Influenza Protection Enhanced by Catalytic Immune‐Adjuvant
Source: Adv Sci (Weinh). 2020 Aug 2;7(18):2000771. doi: 10.1002/advs.202000771 (PMC7509716; doi:10.1002/advs.202000771)
Supplement: Supplementary file 1 — Supporting Information [file ADVS-7-2000771-s001.pdf]

© 2020 Wiley-VCH GmbH

ADVANCED SCIENCE
Open Access

Supporting Information

for *Adv. Sci.,* DOI: 10.1002/advs.202000771

Mucosal Vaccination for Influenza Protection Enhanced by Catalytic Immune-Adjuvant

Tao Qin, Shang Ma, Xinyu Miao, Yan Tang, Dandan Huangfu, Jinyuan Wang, Jing Jiang, Nuo Xu, Yuncong Yin, Sujuan Chen, Xiufan Liu, Yinyan Yin, Daxin Peng,* and Lizeng Gao**

Supporting Information

Mucosal Vaccination for Influenza Protection Enhanced by Catalytic Immune-Adjuvant

Tao Qin[1,4,5,6†], Shang Ma[2†], Xinyu Miao[1], Yan Tang[2], Dandan Huangfu[1], Jinyuan Wang[1], Jing Jiang[2], Nuo Xu,[2] Yuncong Yin[1], Sujuan Chen[1,4,5,6], Xiufan Liu[1,4,5], Yinyan Yin[2,4], Daxin Peng[1,4,5,6]*, and Lizeng Gao[2,3,4]**

Supporting Information

Mucosal Vaccination for Influenza Protection Enhanced by Catalytic Immune-Adjuvant

Tao Qin[1,4,5,6†], Shang Ma[2†], Xinyu Miao[1], Yan Tang[2], Dandan Huangfu[1], Jinyuan Wang[1], Jing Jiang[2], Nuo Xu,[2] Yuncong Yin[1], Sujuan Chen[1,4,5,6], Xiufan Liu[1,4,5], Yinyan Yin[2,4]*, Daxin Peng[1,4,5,6]*, and Lizeng Gao[2,3,4]*

[1]College of Veterinary Medicine, Yangzhou University, Yangzhou, Jiangsu, 225009, PR China.

[2]Institute for Translational medicine, School of Medicine, Yangzhou University, Yangzhou, Jiangsu, 225009, PR China.

[3]CAS Engineering Laboratory for Nanozyme, Institute of Biophysics, Chinese Academy of Sciences, Beijing, 100101, PR China.

[4]Jiangsu Co-Innovation Center for the Prevention and Control of Important Animal Infectious Disease and Zoonoses, Yangzhou, Jiangsu, 225009, PR China.

[5]Joint Laboratory Safety of International Cooperation of Agriculture&Agricultural-Products, Yangzhou, Jiangsu, 225009, PR China.

[6]Jiangsu Research Centre of Engineering and Technology for Prevention and Control of Poultry Disease, Yangzhou, Jiangsu, 225009, PR China.

[†]These authors contributed equally to this work

*Corresponding authors: gaolizeng@ibp.ac.cn (Lizeng Gao), yyyin@yzu.edu.cn (Yinyan Yin), and
pengdx@yzu.edu.cn (Daxin Peng)

Table of contents

Figure S1. Characterizations of CS-IONzyme.

Figure S2. Bio-stability of CS-IONzyme.

Figure S3. Biosafety evaluation of CS-IONzyme *in vivo* and *in vitro*.

Figure S4. The ability of viral adhesion to nasal mucosa after intranasal immunization of CS-IONzyme and H1N1 WIV complexes.

Figure S5. Capture of luminal H1N1 WIV by submucosal DCs.

Figure S6. The number of H1N1 WIV-loaded submucosal DCs after intranasal immunization of CS-IONzyme and H1N1 WIV complexes in mice.

Figure S7. The level of virus uptake by DCs *in vitro*.

Figure S8. CCL20 and CCL5 expression by nasal epithelial cells after intranasal immunization of CS-IONzyme and H1N1 WIV complexes in mice.

Figure S9. The ability of DC recruitment into the submucosa after CCL20 neutralizing antibody pretreatment *in vivo*.

Figure S10. CCR7 expression and DCs migration ability *in vitro*.

Figure S11. The number of H1N1 WIV-loaded DCs that migrated into the cervical lymph nodes *in vivo.*

Figure S12. TLRs activation of nasal ECs by CS-IONzyme *in vivo*.

Figure S13. Suppression effects of TLR2 and TLR4 inhibitors *in vivo*.

Figure S14. The expression of CCL20 on the nasal epithelial cells after the pretreatment of TLR2 and TLR4 inhibitors *in vivo*.

Figure S15. The number of submucosal DCs after the pretreatment of TLR2 and TLR4

inhibitors *in vivo*.

Figure S16. The evaluation of DCs maturation *in vitro.*

Figure S17. The proliferation and ROS levels of splenic lymphocytes by CS-IONzyme *in vitro*.

Figure S18. TEM observation of uptake and distribution of IONzyme in DCs *in vitro*.

Figure S19. CLSM observation of uptake and distribution of CS-IONzyme in DCs *in vitro*.

Figure S20. ROS production by CS-IONzyme in DCs *in vitro*.

Figure S21. ROS generation in lysosome by CS-IONzyme in DCs *in vitro*.

Figure S22. DCs maturation by CS-IONzyme treatment after blocking the ROS by NAC.

Figure S23. The expression of activation marker on splenocytes.

Figure S24. The percentages of $CD3^+$ $CD4^+$ and $CD3^+$ $CD8^+$ splenic T cells.

Figure S25. The pathological and histopathological change of murine lungs post challenge.

Figure S26. The pathological change of murine spleens post challenge.

Figure S27. The expression of TLR2/4 on DCs *in vitro*.

Table S1. List of antibodies used for flow cytometry.

Table S2. List of antibodies used for immunofluorescence.

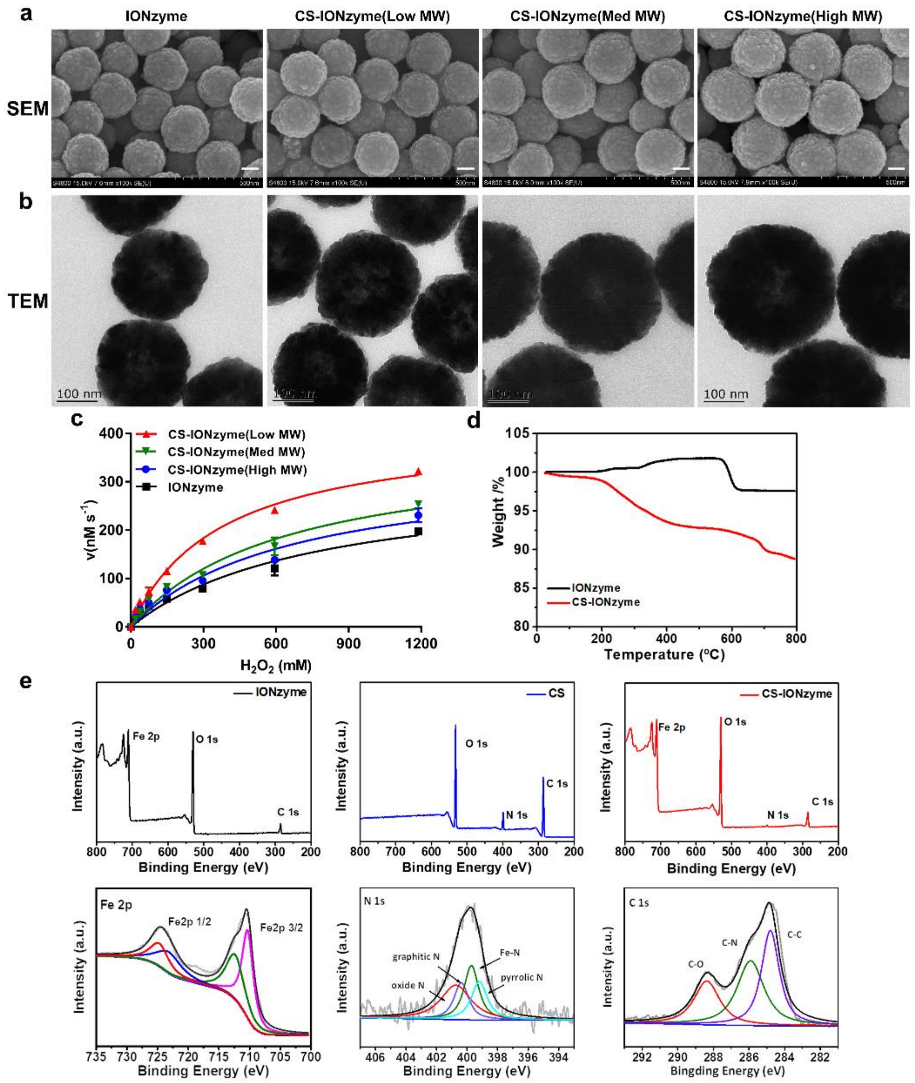

Figure S1. Characterizations of CS-IONzyme. a) The SEM images of the prepared CS-IONzyme. Scale bar: 100 nm. b) The TEM micrograph of the prepared CS-IONzyme. Scale bar: 100 nm. c) Michaelis-Menten kinetics for peroxidase-like activity of CS-IONzyme towards H_2O_2. The reaction was conducted in NaOAc buffer (0.1 M, pH 4.5) containing 10 μg CS-IONzyme and 0.8 mM TMB with variable H_2O_2 concentrations. Data shown represent the means±s.d. of three independent experiments. d) Thermal gravimetric analysis (TGA) of the product of CS (low molecular weight, 50-190 KDa)-IONzyme. e) X-ray photoelectron spectroscopy spectra of IONzyme modified with CS (50-190 KDa). Top panel, XPS spectra of IONzyme, CS, and CS-IONzyme. Down panel, Fe 2p, N 1s, and C 1s of CS-IONzyme from up panel were further analyzed. Representative results are shown.

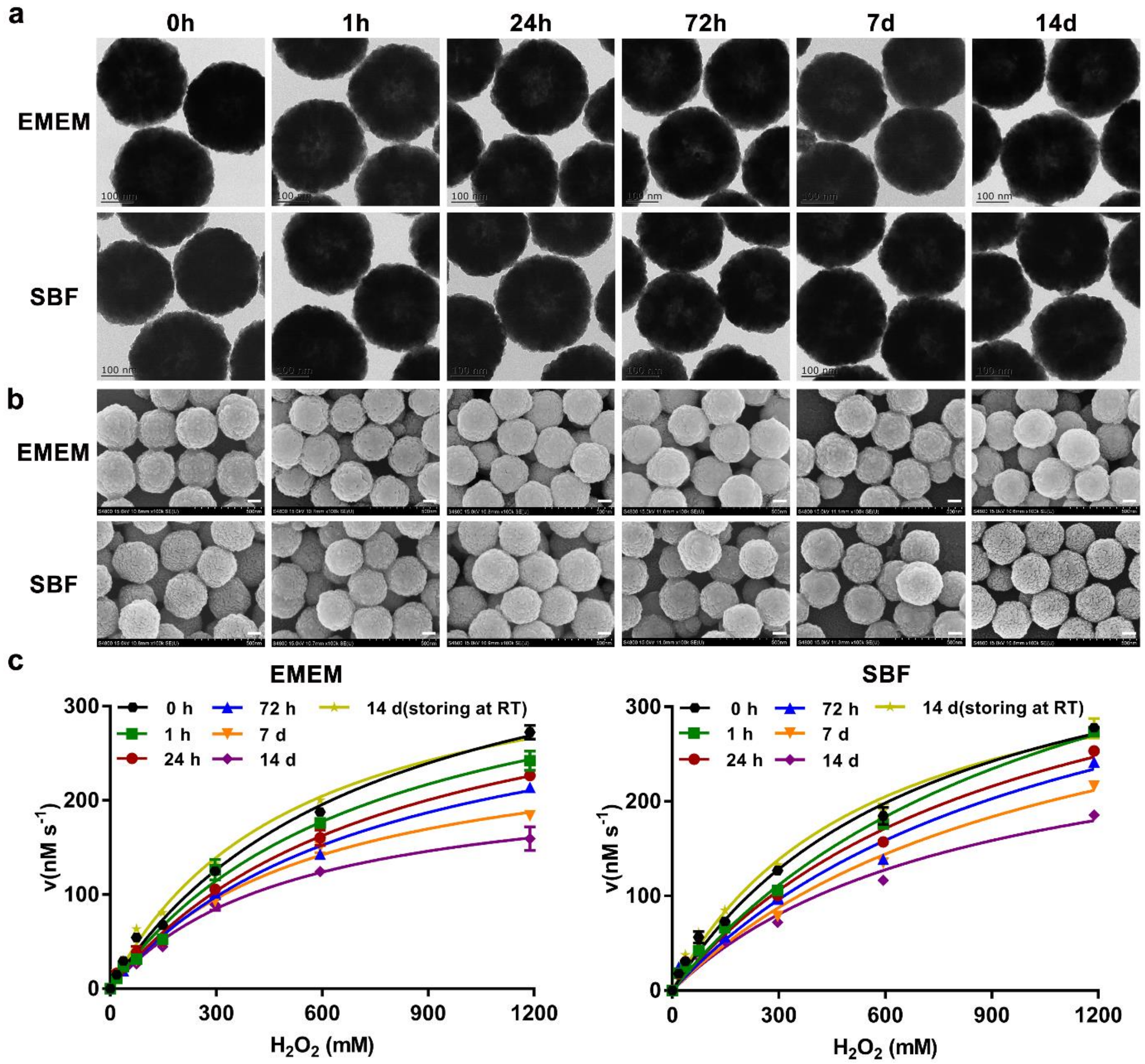

Figure S2. Bio-stability of CS-IONzyme. CS-IONzyme were tested in the simulated body fluid (SBF) and cell culture medium (EMEM) completed with 10% of fetal bovine serum at 37℃ for 0 h, 1 h, 24 h, 72 h, 7 d, and 14 d. a-b) The TEM and SEM images of CS-IONzyme at different time points. Representative images are shown. Scale bar: 100 nm. c) Michaelis-Menten kinetics for peroxidase-like activity of CS-IONzyme with different treatment at different time point towards H_2O_2. The reaction was conducted in NaOAc buffer (0.1 M, pH 4.5) containing 10 μg CS-IONzyme and 0.8 mM TMB with variable H_2O_2 concentrations. Data shown represent the means±s.d. of three replicates. Storing at RT means that CS-IONzyme without SBF or EMEM treatment were stored at room temperature for 14 d.

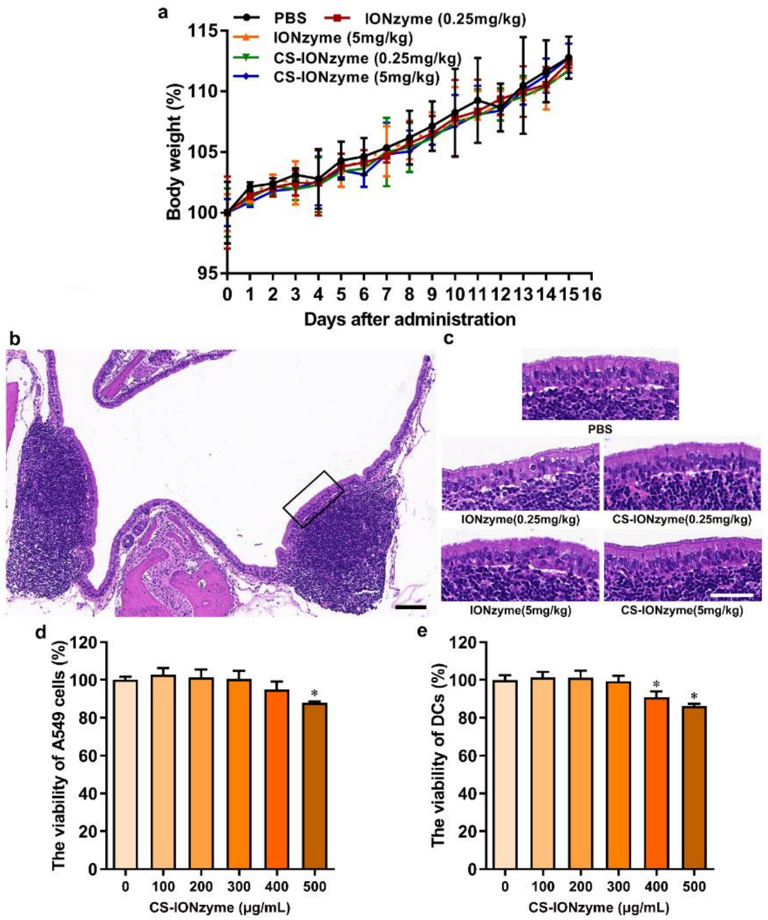

Figure S3. Biosafety evaluation of CS-IONzyme *in vivo* and *in vitro*. IONzyme or CS-IONzyme with different concentrations was intranasally dropped in mice. a) The data represent the change of body weight of each group (n=10/group). b) Representative histopathological changes in H&E (hematoxylin and eosin)-stained nasal tissues at 3 day post after treatment. c) The enlargements of the regions in the black frame (b) show the epithelium located in the surface of nasal mucosa. Results are from one representative experiment of two performed. Bars: (b) 100 μm; (c) 50 μm. d-e) The cytotoxicity test of CS-IONzyme with different doses was performed in the A549 epithelial cells and DCs, and detected by using the CCK-8 assay. The data are presented as means±s.d. of three replicates and are representative of three independent experiments. Statistical significance is assessed by unpaired Student's two-sided *t*-test to compare CS-IONzyme (0 μg/mL). **P*<0.05.

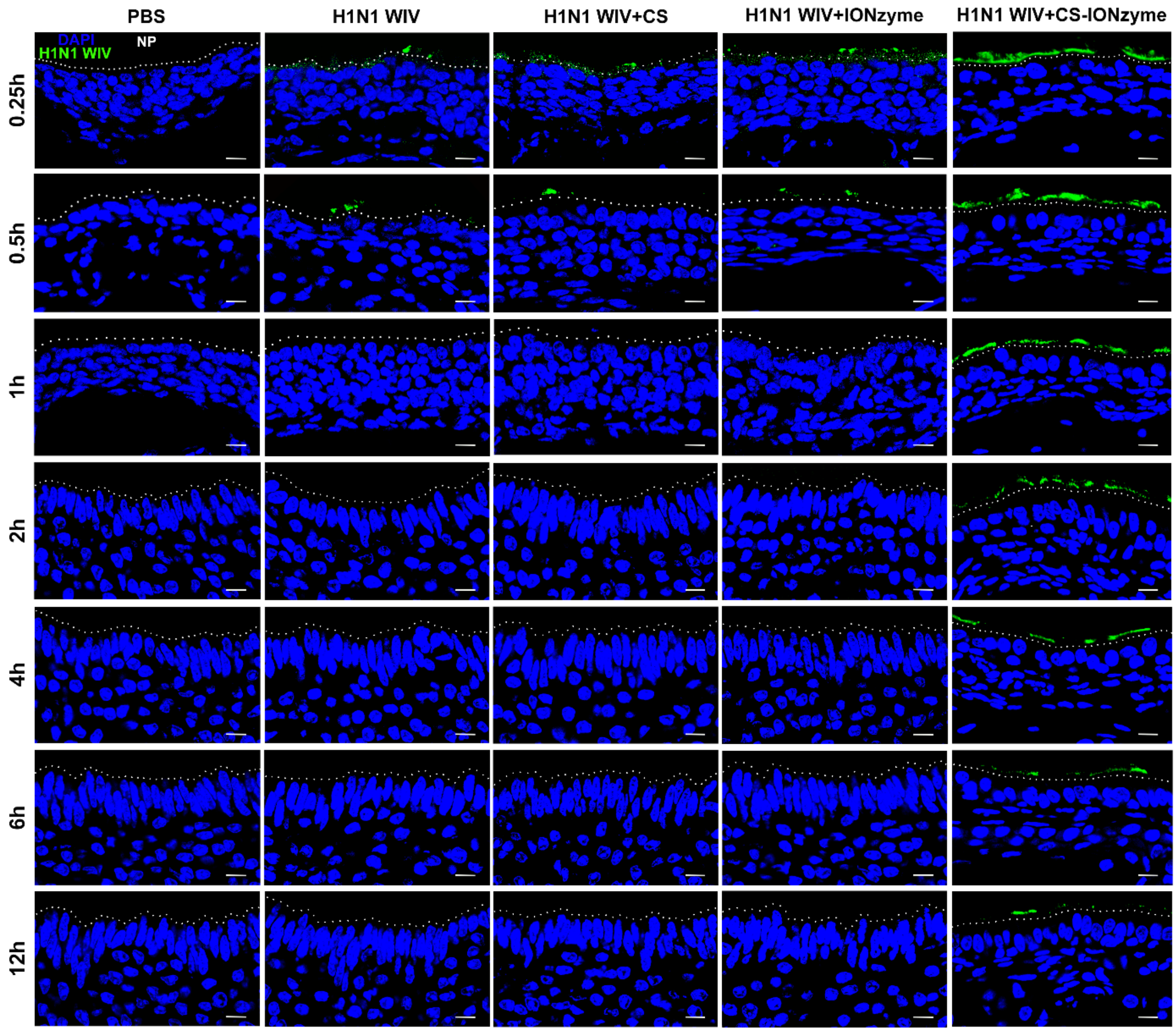

Figure S4. The ability of viral adhesion to nasal mucosa after intranasal immunization of CS-IONzyme and H1N1 WIV complexes. Mice (n=6/group) were nasally immunized with either PBS or vaccines for 0.25, 0.5, 1, 2, 4, 6, and 12 h, and then noses were collected for frozen sections. Frozen sections of nasal tissues were observed for evaluating the adhesion levels of H1N1 WIV to nasal mucosa by using confocal laser scanning microscopy (CLSM). H1N1 WIV (DyLight 488; green), and Nuclei (DAPI; blue). Results are from one representative experiment of three performed. Bars: 10 μm.

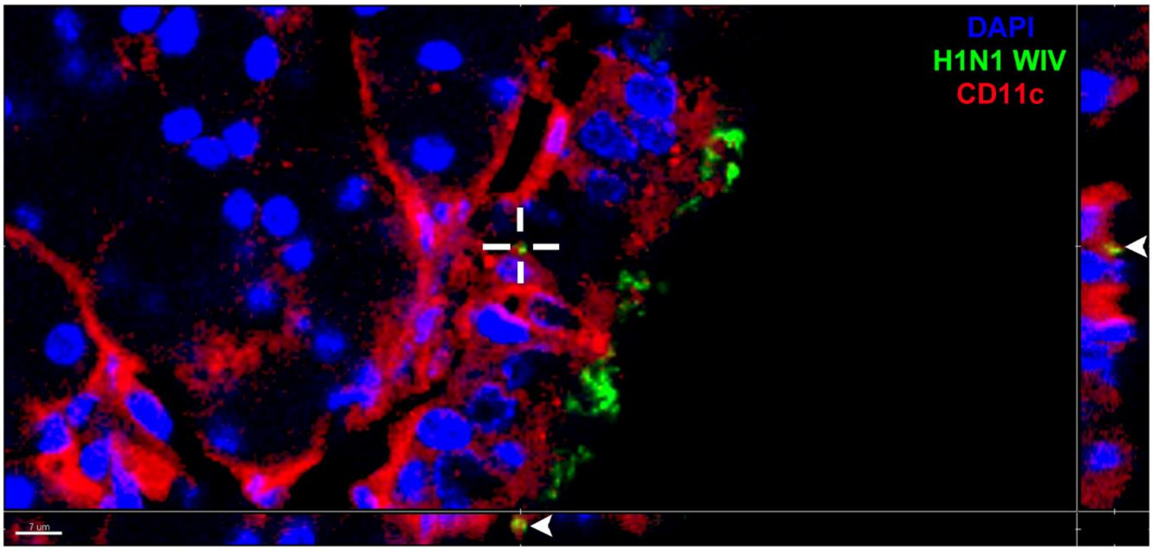

Figure S5. Capture of luminal H1N1 WIV by submucosal DCs. Immunofluorescence stain of the murine nasal cavity (n=10) after nasally administering of CS-IONzyme and H1N1 WIV complexes for 0.5 h. Cross-sectional images of representative fields obtained with Imaris 7.2 software, showing virus uptake by submucosal DCs (arrows), and virus internalization by the bodies of DCs (cross hairs). Nuclei (DAPI; blue), H1N1 WIV (DyLight 488; green), CD11c (Alexa Fluor 649; red). Results are from one representative experiment of two performed. Bar: 7 μm.

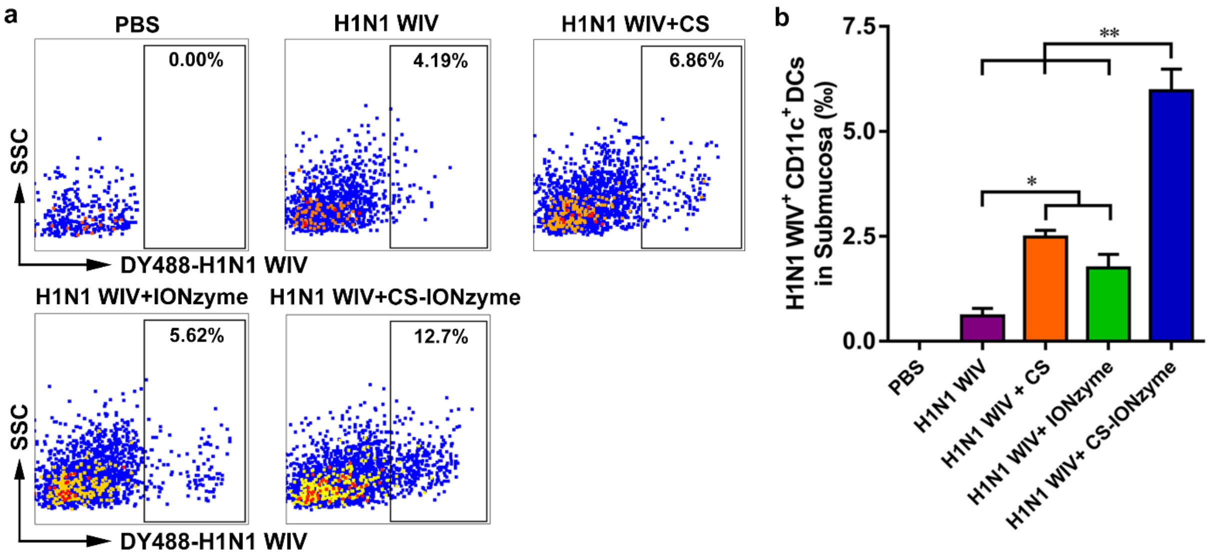

Figure S6. The number of H1N1 WIV-loaded submucosal DCs after intranasal immunization of CS-IONzyme and H1N1 WIV complexes in mice. Mice (n=6/group) were nasally dropped CS-IONzyme and H1N1 WIV complexes and other different control groups for 0.5 h. a) H1N1 WIV-loaded submucosal DCs (CD11c^{+} H1N1 WIV^{+}) were analyzed by FCM. b) Quantitative detection of viral capture by submucosal DCs as shown in panel a. Statistical significance is assessed by One-way ANOVA analysis to compare the results between different groups. Results are from one representative experiment of three performed. Data shown are the means±s.d. **P*<0.05; ***P*<0.01.

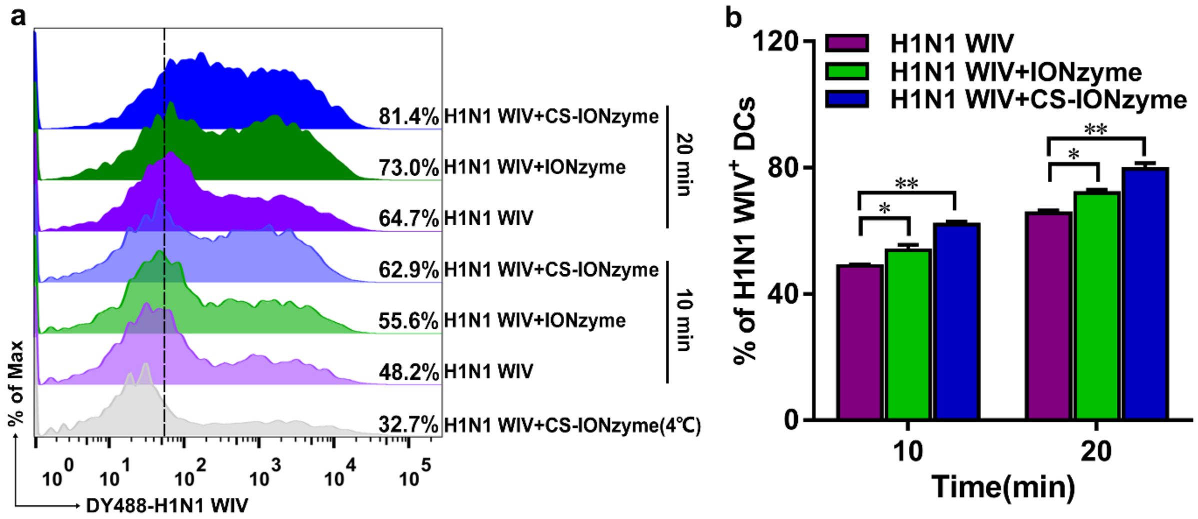

Figure S7. The level of virus uptake by DCs *in vitro*. DCs were incubated with DyLight 488-labeled H1N1 WIV plus IONzyme/CS-IONzyme or not for 10 min or 20 min. 4℃ control was also added to exclude adhesion. a) FCM analysis of viral uptake by DCs. b) Quantification of the FCM results as shown in panel a. Data shown represent the means±s.d of three replicates and are representative of three independent experiments. Statistical significance is assessed by unpaired Student's two-sided *t*-test to compare H1N1 WIV group. **P*<0.05, ***P*<0.01.

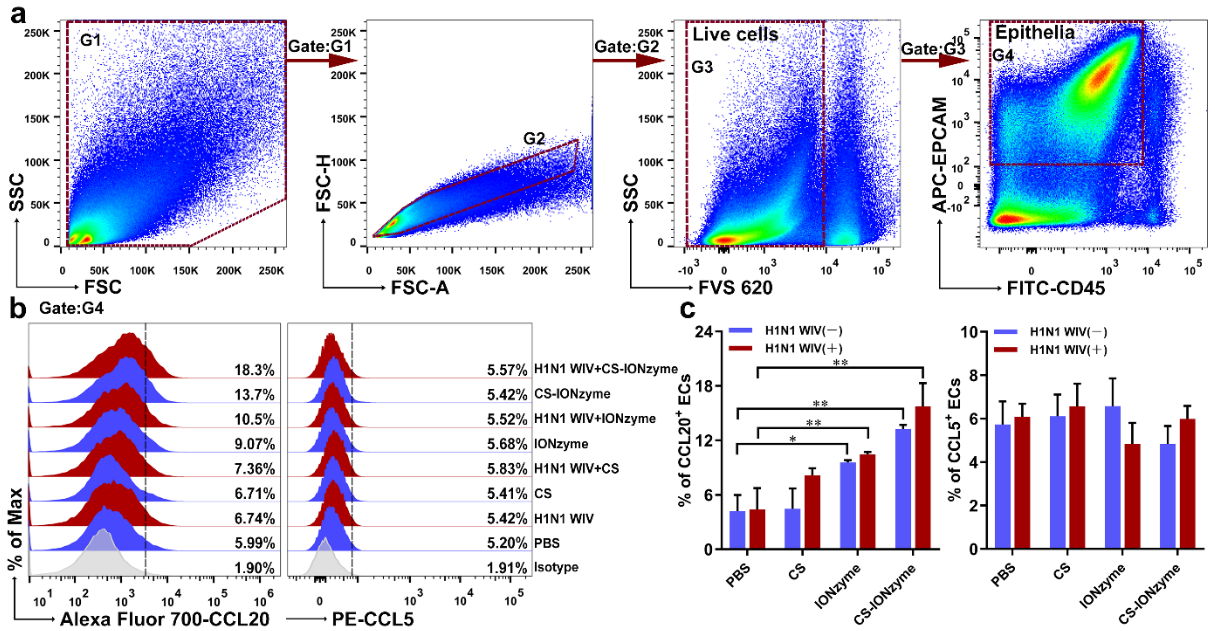

Figure S8. CCL20 and CCL5 expression by nasal epithelial cells after intranasal immunization of CS-IONzyme and H1N1 WIV complexes in mice. Mice (n=6/group) were nasally dropped CS-IONzyme and H1N1 WIV complexes and other different control groups for 0.5 h. a) For FCM analysis, nasal-associated lymphoid tissues (NALTs) were removed from noses, and the individual cells isolated from nasal cavity were firstly gated to remove the synechia cells (G2), and then gated to choose live cells (G3). Furthermore, epithelial cells were gated based on EPCAM^{+} CD45^{-} (G4). b) CCL20 and CCL5 expression in the gate of epithelial cells (G4) were analyzed. c) Quantification of the FCM results as shown in panel b. Results are from one representative experiment of two performed. Data shown are the means±s.d. Statistical significance is assessed by One-way ANOVA analysis to compare the results between different groups. *P<0.05; **P<0.01.

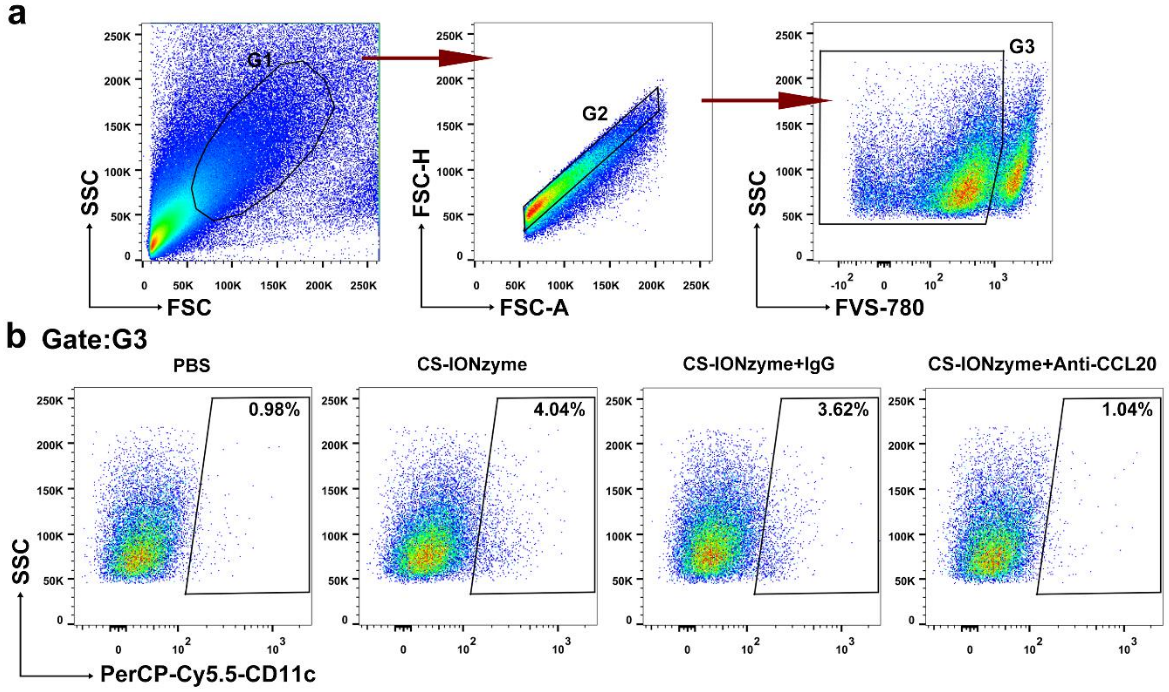

Figure S9. The ability of DC recruitment into the submucosa after CCL20 neutralizing antibody pretreatment *in vivo*. CCL20 neutralizing antibody (100 μg/mouse), or rabbit IgG control was administered to mice (n=6/group) intraperitoneally for 2 h, and then CS-IONzyme was intranasally dropped in the mice for 0.5 h. a) For FCM analysis, nasal-associated lymphoid tissues (NALTs) were removed from noses, and the individual cells isolated from nasal cavity were firstly gated to remove the synechia cells (G2), and then gated to choose live cells (G3). b) DCs were further gated based on CD11c^{+}. Results are from one representative experiment of two performed.

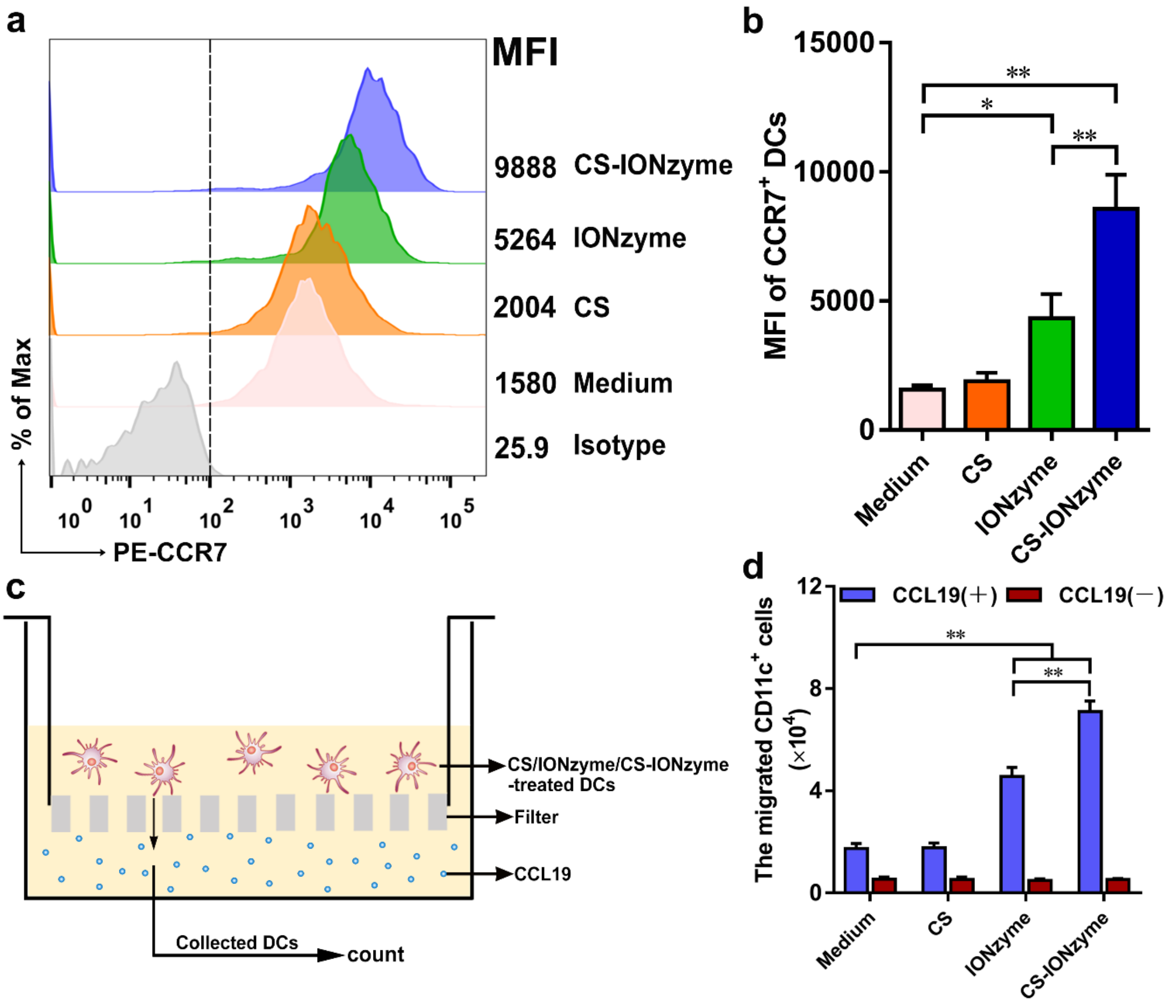

Figure S10. CCR7 expression and DCs migration ability *in vitro*. Murine DCs were incubated by CS, IONzyme, and CS-IONzyme for 24 h. a-b) FCM analysis of CCR7 expression. c-d) DCs were then seeded into the upper wells of a 24-well transwell chamber, and CCL19 (200 ng/mL) was added in lower chamber. After 4 h, the number of cells that transferred from the upper to the lower wells was counted by FCM. The spontaneous migration of cells (absence of CCL19) was also shown. All of the data are presented as means±s.d. of three replicates and are representative of three independent experiments. Statistical significance is assessed by One-way ANOVA analysis to compare the results between different groups. *P<0.05; **P<0.01.

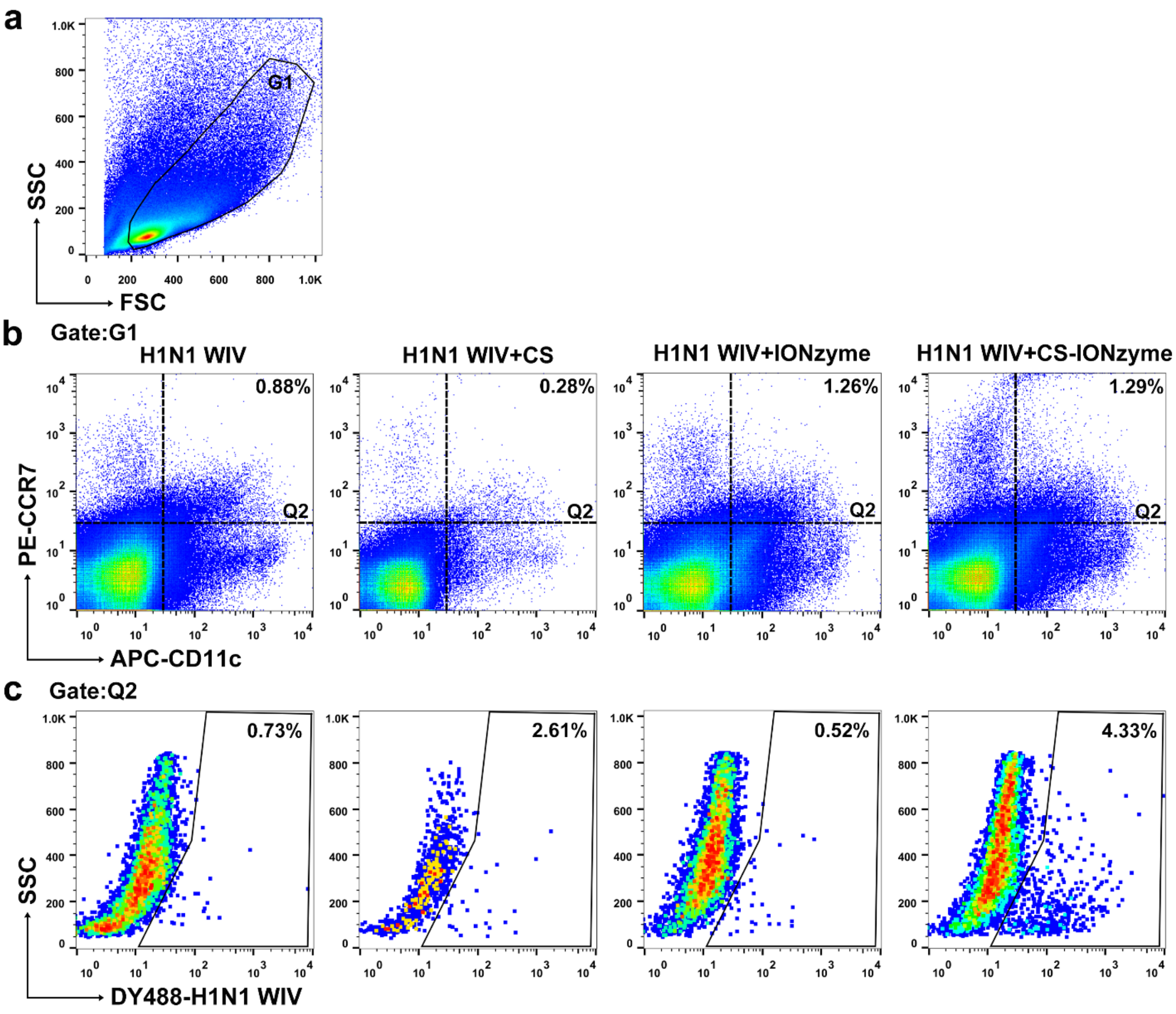

Figure S11. The number of H1N1 WIV-loaded DCs that migrated into the cervical lymph nodes *in vivo*. Mice (n=6/group) were nasally dropped CS-IONzyme and H1N1 WIV complexes and other different control groups for 2 h. a) Cervical lymph nodes (CLNs) cells were isolated and gated by FCM. b) Migrated DCs were gated from G1 based on CD11c^{+}CCR7^{+}. c) Gated cells were further selected from Q2 based on the uptake of DY488-H1N1 WIV. Results are from one representative experiment of two performed.

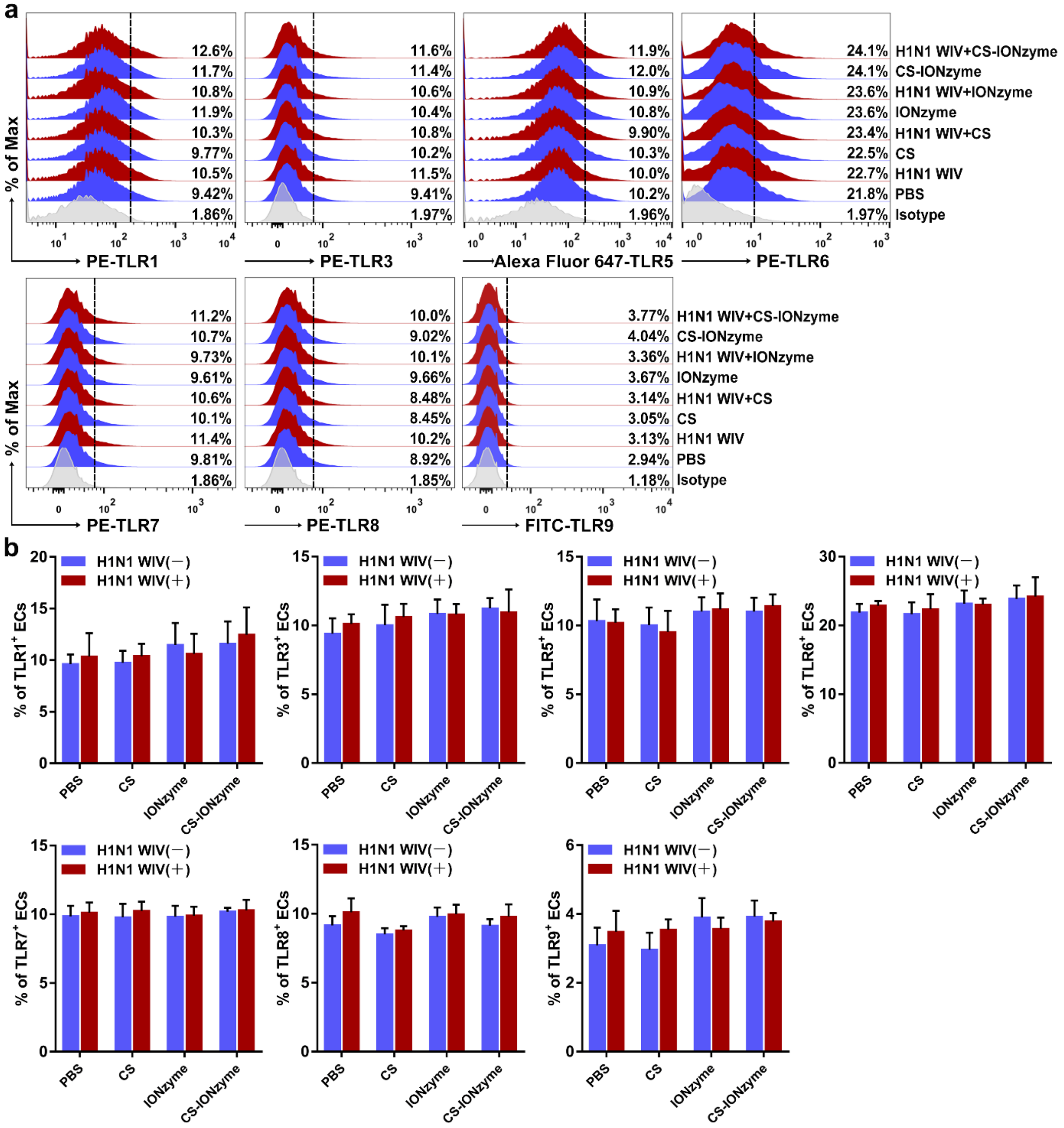

Figure S12. TLRs Activation of nasal ECs by CS-IONzyme *in vivo*. a) FCM analysis of TLR1-9 except TLR2 and TLR4 on the nasal ECs in mice (n=6/group) after nasally administering of CS-IONzyme and H1N1 WIV complexes for 0.5 h. The TLR^{+} cells were gated from EPCAM^{+} CD45^{-} cells. b) Quantification of the FCM results as shown in panel a. Data shown are the means±s.d. Results are from one representative experiment of three performed. Statistical significance is assessed by One-way ANOVA analysis to compare the results between different groups.

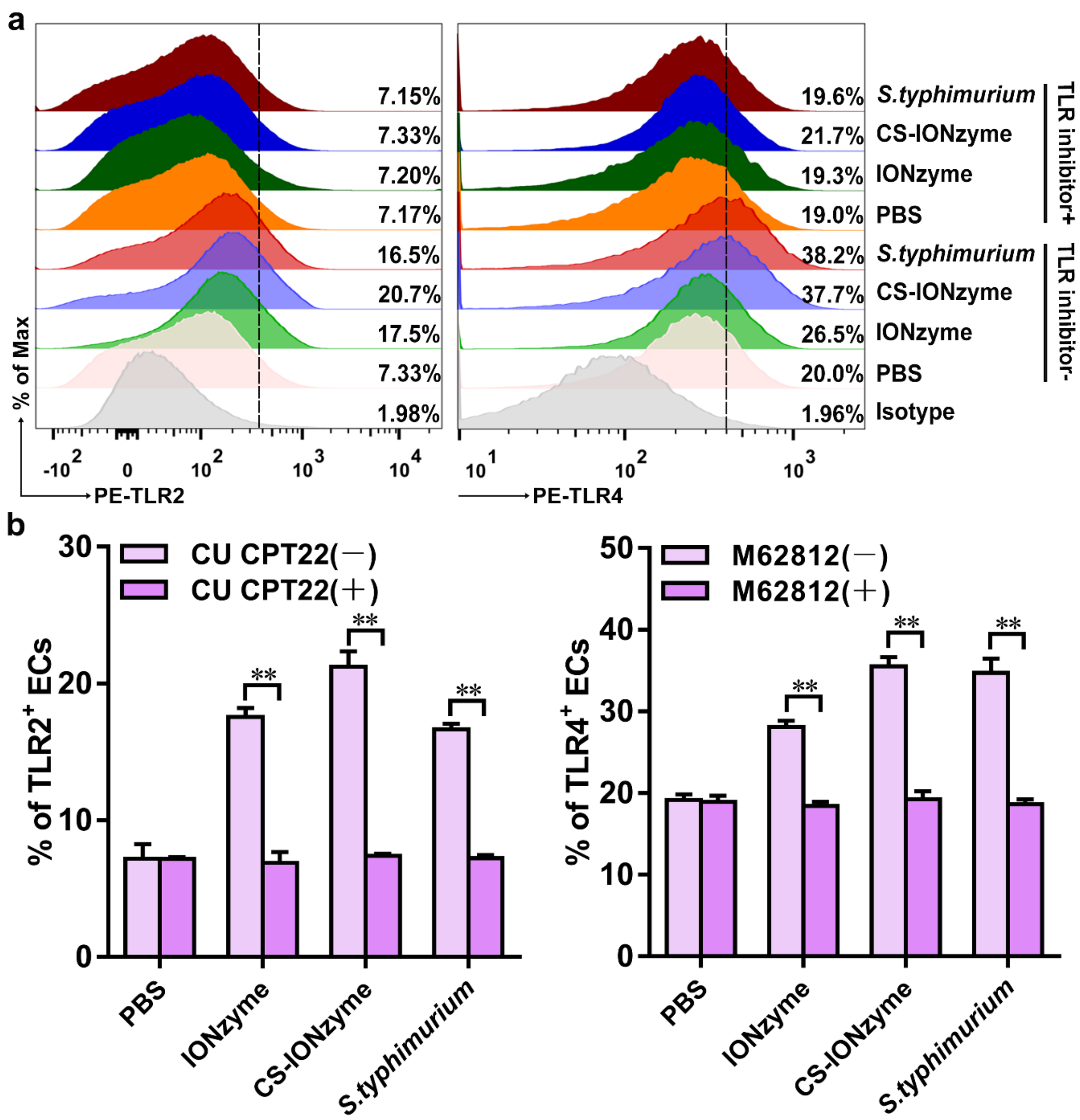

Figure S13. Suppression effects of TLR2 and TLR4 inhibitors *in vivo*. CU CPT22 inhibitor (3 mg/kg) against TLR2, or M62812 inhibitor (20 mg/kg) against TLR4 was administered to mice (n=6/group) intraperitoneally 4 h or intravenously 1.5 h respectively before nasally administering of IONzyme, CS-IONzyme, *S. typhimurium* (positive control). a) FCM analysis of TLR2 and TLR4 on the nasal ECs. The TLR$^+$ cells were gated from EPCAM$^+$ CD45$^-$ cells. b) Quantification of the FCM results as shown in panel a. Data shown are the means±s.d. Results are from one representative experiment of two performed. Statistical significance is assessed by unpaired Student's two-sided *t*-test to compare between the two groups. $^{**}P$<0.01.

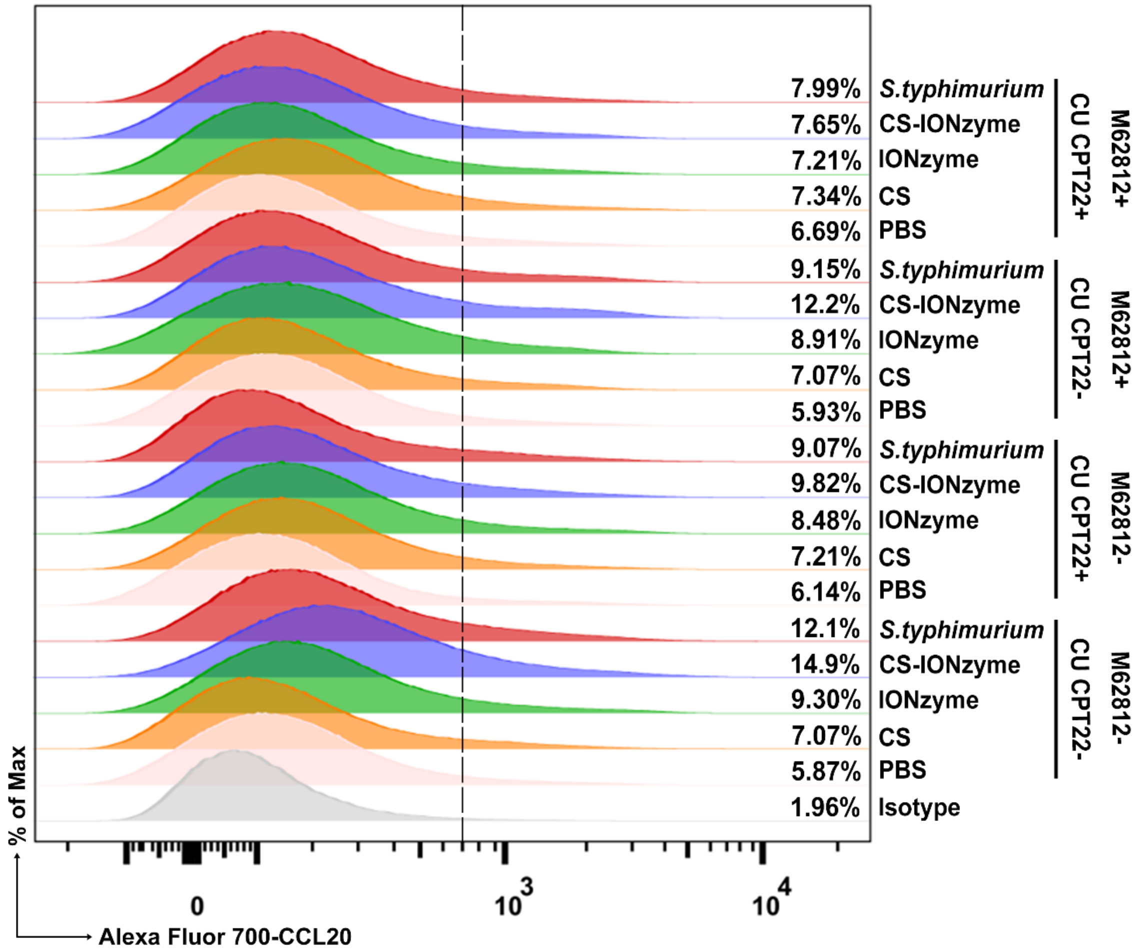

Figure S14. The expression of CCL20 on the nasal epithelial cells after the pretreatment of TLR2 and TLR4 inhibitors *in vivo*. CU CPT22 inhibitor (3 mg/kg) against TLR2, or M62812 inhibitor (20 mg/kg) against TLR4 was administered to mice (n=6/group) intraperitoneally 4 h or intravenously 1.5 h respectively before nasally administering of IONzyme, CS-IONzyme, *S. typhimurium* (positive control). FCM analysis of $CCL20^+$ nasal ECs. The $CCL20^+$ ECs were gated from $EPCAM^+$ $CD45^-$ cells of nasal tissues. Results are from one representative experiment of three performed.

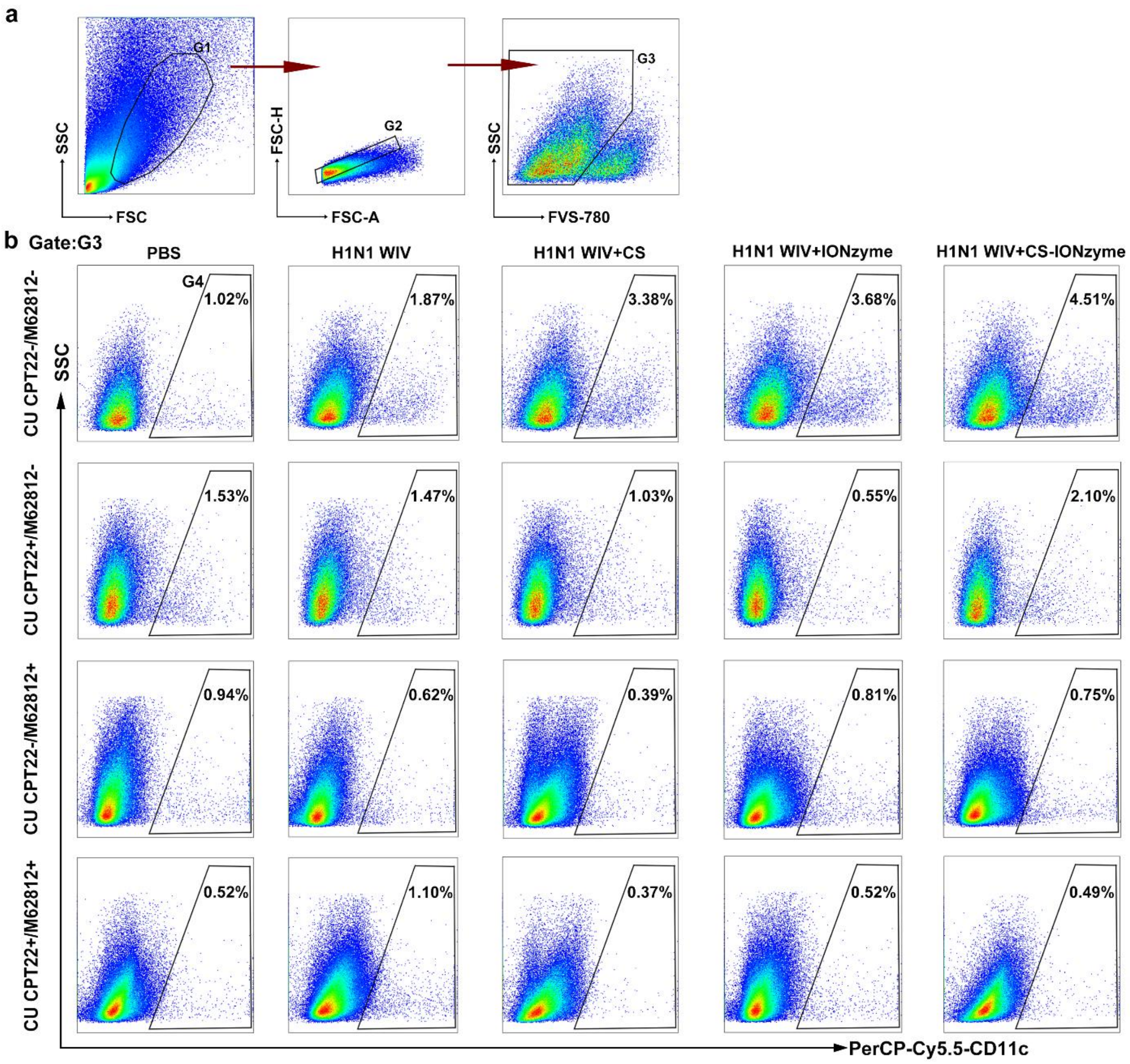

Figure S15. The number of submucosal DCs after the pretreatment of TLR2 and TLR4 inhibitors *in vivo*. CU CPT22 inhibitor (3 mg/kg) against TLR2, or M62812 inhibitor (20 mg/kg) against TLR4 was administered to mice (n=6/group) intraperitoneally 4 h or intravenously 1.5 h respectively before nasally administering of CS-IONzyme and H1N1 WIV complexes for 0.5 h. a) For FCM analysis, NALTs were removed from noses, and the individual cells isolated from nasal tissues were firstly gated to remove the synechia cells (G2), and then gated to choose live cells (G3). b) Submucosal DCs were gated from G3 based on the CD11c$^+$. Results are from one representative experiment of three performed.

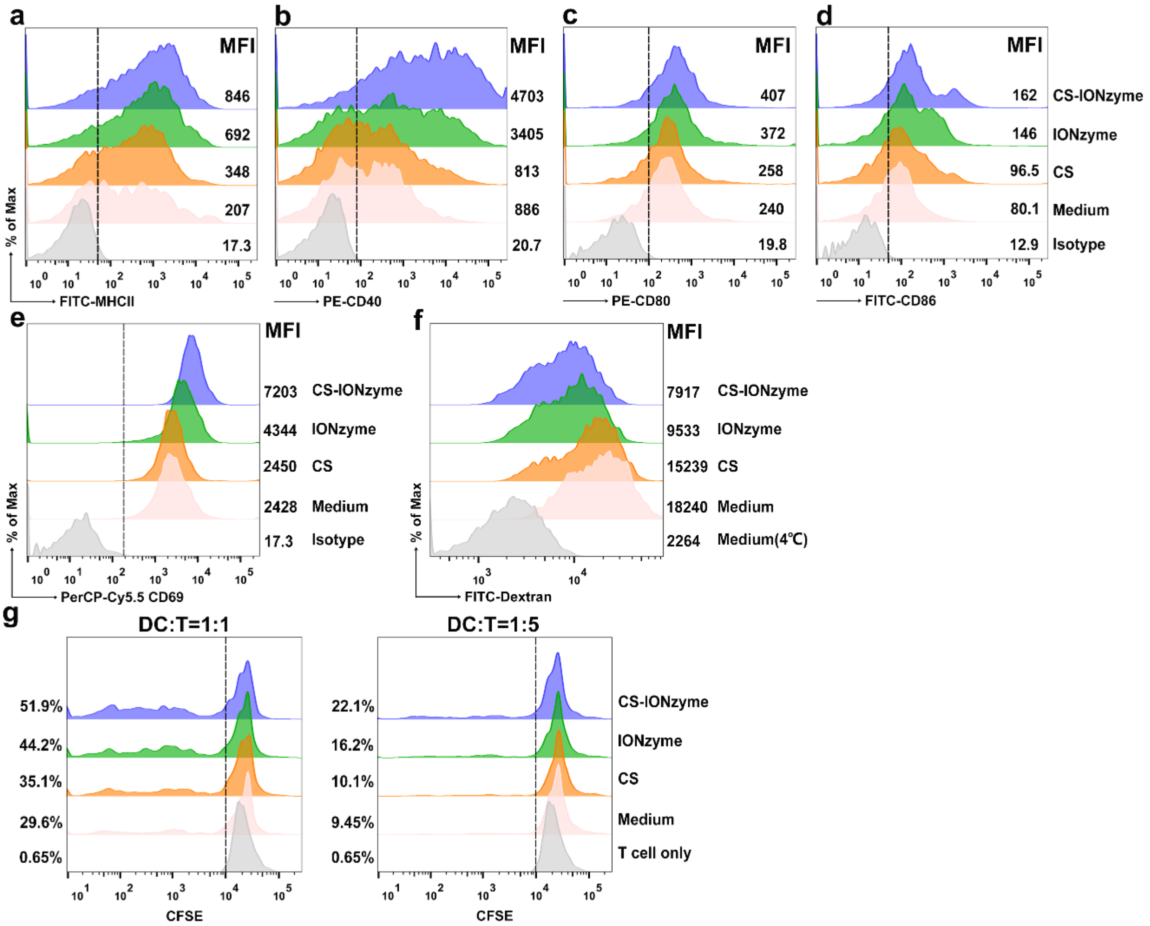

Figure S16. The evaluation of DCs maturation *in vitro*. a-d) The expressions of phenotypic markers on DCs *in vitro*. After stimulation for 24 h with CS-IONzyme or other controls, the expressions of phenotypic markers on DCs, including MHCII, CD40, CD80, and CD86 were analyzed by FCM. e) The expression of activation marker CD69 on DCs was analyzed by FCM *in vitro*. f) The endocytosis ability of DCs after CS-IONzyme treatment *in vitro*. After stimulation for 24 h with CS-IONzyme or other controls, the treated DCs were incubated at 37℃ for 30 min with 1 mg/mL FITC-Dextran. After incubation, DCs were washed twice with cold washing PBS and analyzed by FCM. Parallel experiments were performed at 4℃ to determine the nonspecific binding. g) Allogenic mixed lymphocyte reaction *in vitro*. After stimulation for 24 h with CS-IONzyme or other controls, the treated DCs were used in two graded cell numbers (DC/T-cell ratios: 1:1 and 1:5) to stimulate Carboxyfluorescein Succinimidyl Ester (CFSE)-labeled naive $CD4^+$ allogeneic T cells (5×10^5 responder cells per well). After 5 days, proliferation was detected by FCM. Images are from a representative experiment out of three.

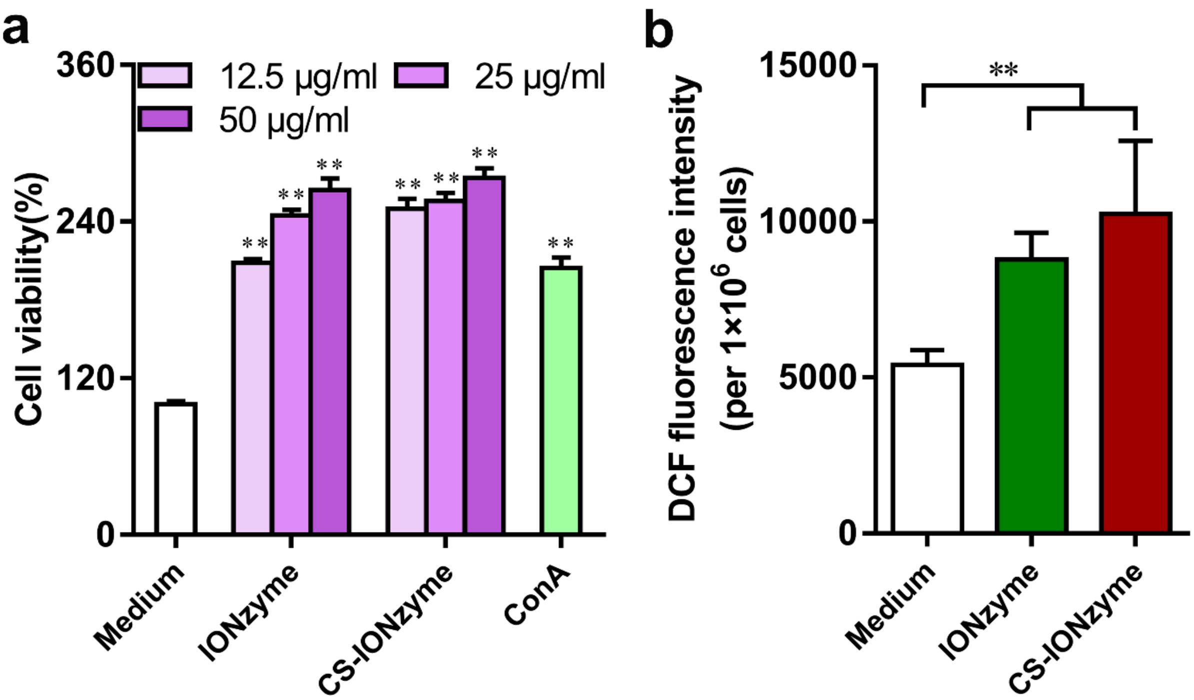

Figure S17. The proliferation and ROS levels of splenic lymphocytes by CS-IONzyme *in vitro*. Lymphocytes were separated from spleens of BALB/c mice, a) IONzyme, CS-IONzyme, and ConA (positive control, 5 μg/mL) were incubated with lymphocytes in 96-well plates for 3 days and then the proliferative level was detected by using the CCK-8 assay. b) Lymphocytes (1×10^6 cells/mL) were treated by IONzyme (50 μg/mL) or CS-IONzyme (50 μg/mL) for 24 h, and then cells were stained with DCFH-DA and analyzed by fluorescence microplate reader for ROS detection. All of the data are presented as means±s.d. of three replicates and are representative of three independent experiments. Statistical significance is assessed by unpaired Student's two-sided *t*-test to compare the medium group. **P<0.01.

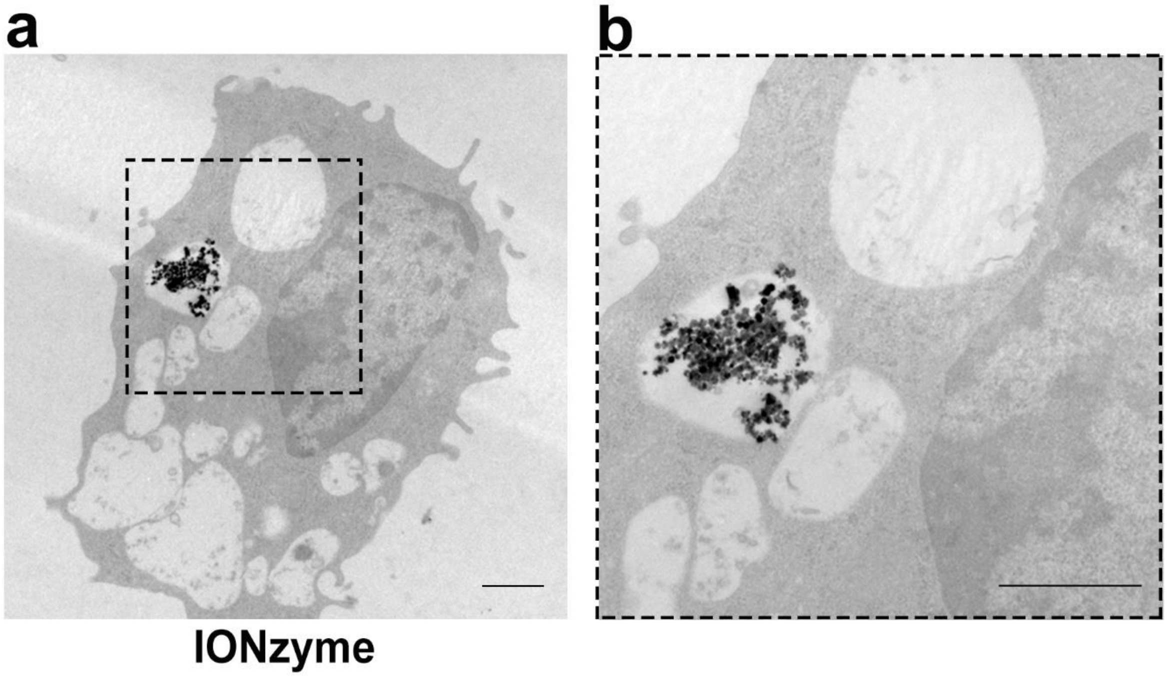

Figure S18. TEM observation of uptake and distribution of IONzyme in DCs *in vitro*. a-b), TEM image of the internalization of IONzyme in the lysosome of DCs. An enlargement of the region in the black frame in panel a (scale bar: 1 μm) shows in panel b (scale bar: 1 μm). The picture is presented as one of three independent experiments.

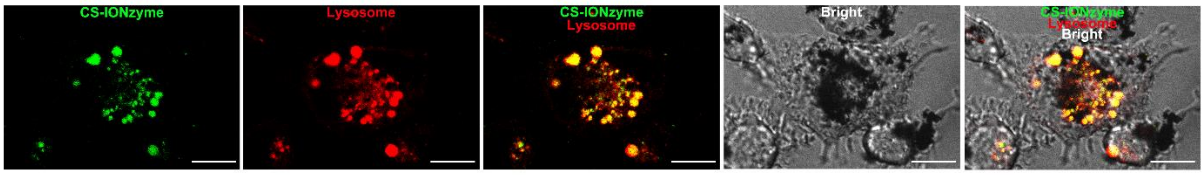

Figure S19. CLSM observation of uptake and distribution of CS-IONzyme in DCs *in vitro*. After stimulation for 1 h with 100 μg/mL FITC-labeled CS-IONzyme, Lyso-Tracker Red-loaded DCs were observed by CLSM. CS-IONzyme (green), lysosome (red). The picture is presented as one of three independent experiments. Scale bar: 10 μm.

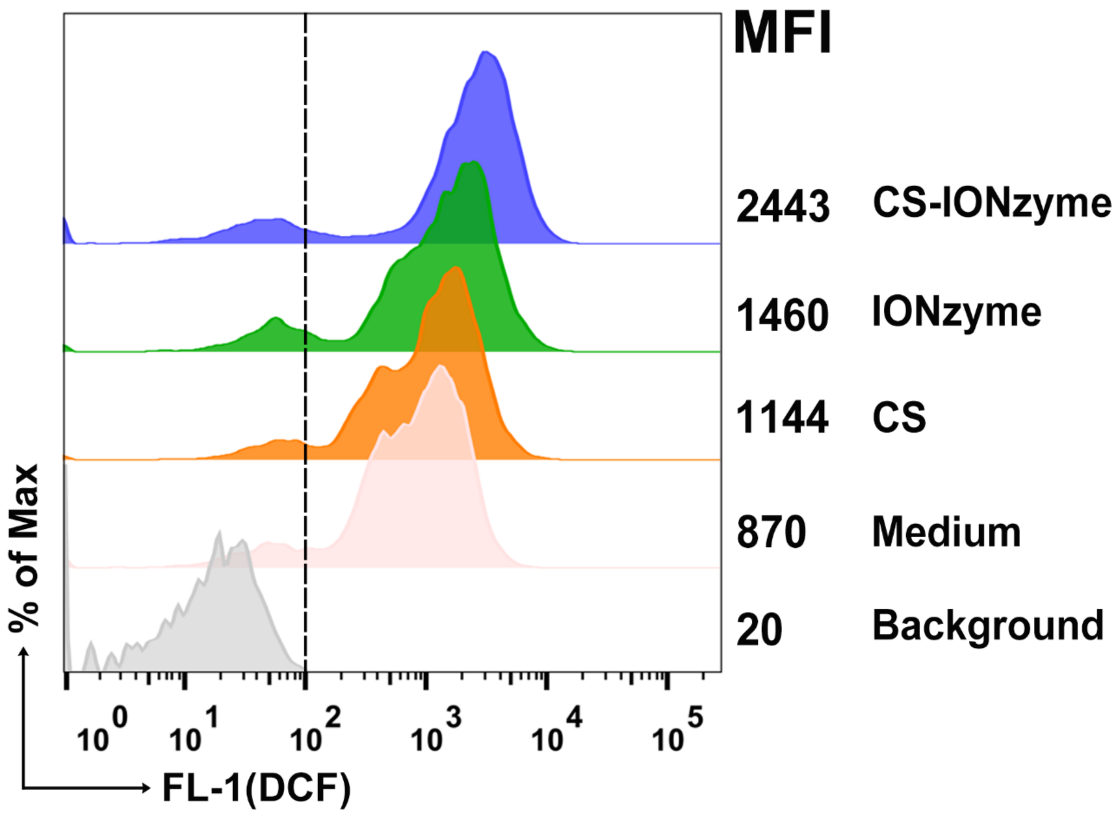

Figure S20. ROS production by CS-IONzyme in DCs *in vitro*. After stimulation for 24 h with CS-IONzyme or other controls, DCs were stained with DCFH-DA and analyzed by FCM for ROS detection. The picture is presented as one of three independent experiments.

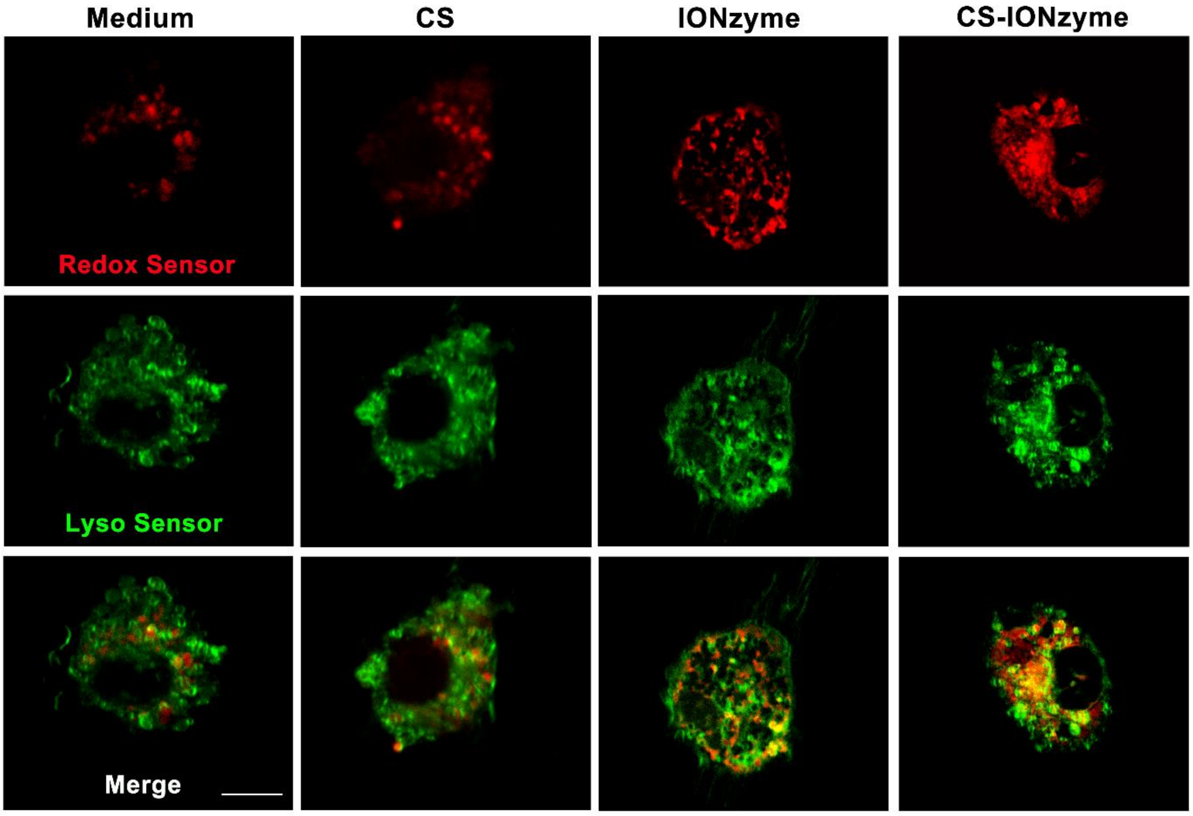

Figure S21. ROS generation in lysosome by CS-IONzyme in DCs *in vitro*. After stimulation for 24 h with CS-IONzyme or other controls, DCs were stained with RedoxSensor^{TM} Red CC-1 to detect intracellular ROS and LysoSensor Green as a lysosome marker. Colocalization (yellow) indicates a ROS generation in lysosomes. The picture is presented as one of three independent experiments. Scale bar: 10 μm.

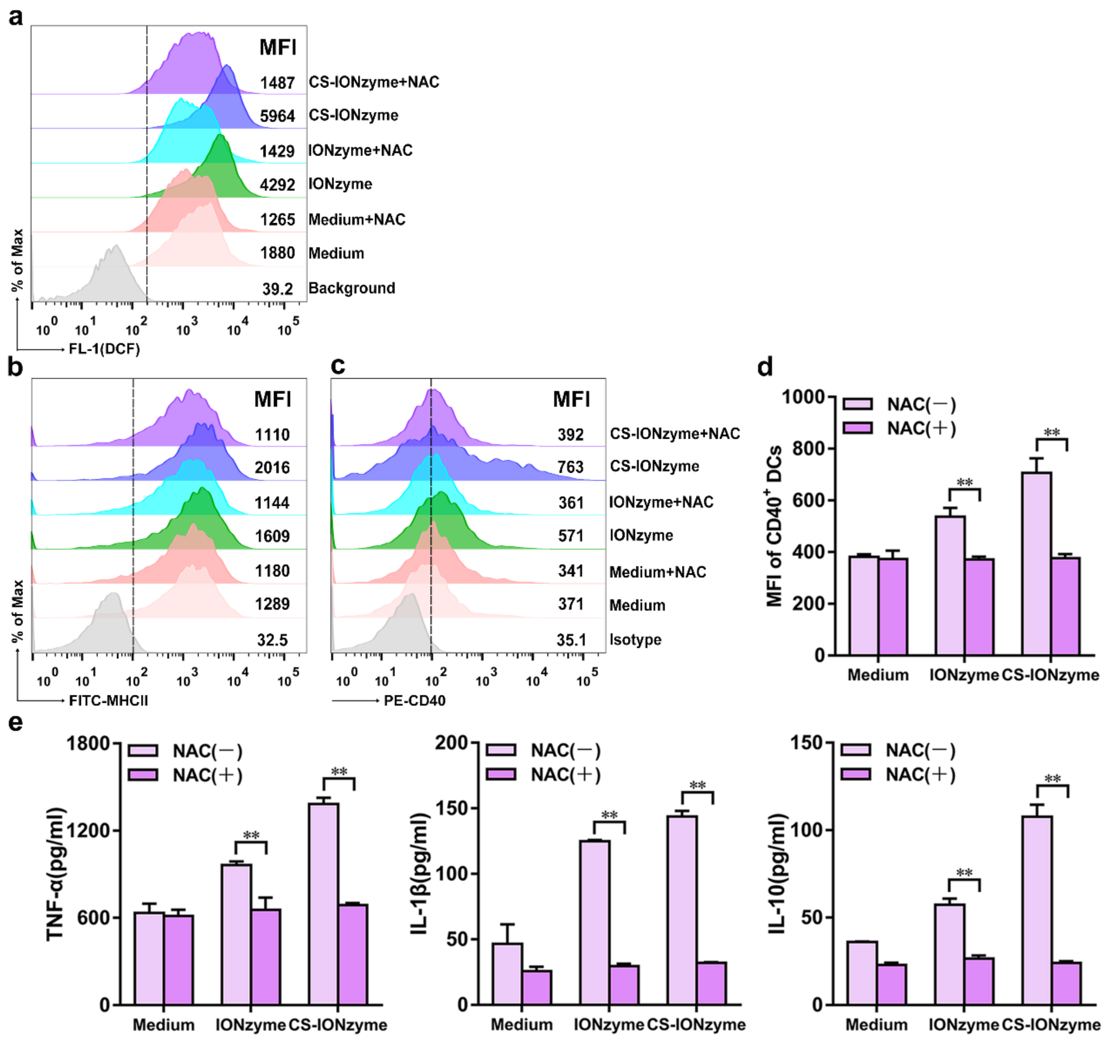

Figure S22. DCs maturation by CS-IONzyme treatment after blocking the ROS by NAC. a) The block of ROS by NAC in CS-IONzyme-treated DCs. DCs were pretreated with ROS scavengers (NAC, 3 mM) or not for 1 h, and then were stimulated for 24 h with CS-IONzyme or other groups. DCs were stained with DCFH-DA for ROS detection and analyzed by FCM. b-d) The expression of MHCII and CD40 on the NAC-pretreated DCs after CS-IONzyme incubation. MHCII and CD40 on DCs were detected and analyzed by FCM. e) The secretion of cytokines from the NAC-pretreated DCs after CS-IONzyme incubation. TNF-α, IL-1β, and IL-10, were detected by ELISA kit. All of the data are presented as means±s.d. of three replicates and are representative of three independent experiments. Unpaired Student's two-sided *t*-test is employed to compare between the two groups. $^{**}P<0.01$.

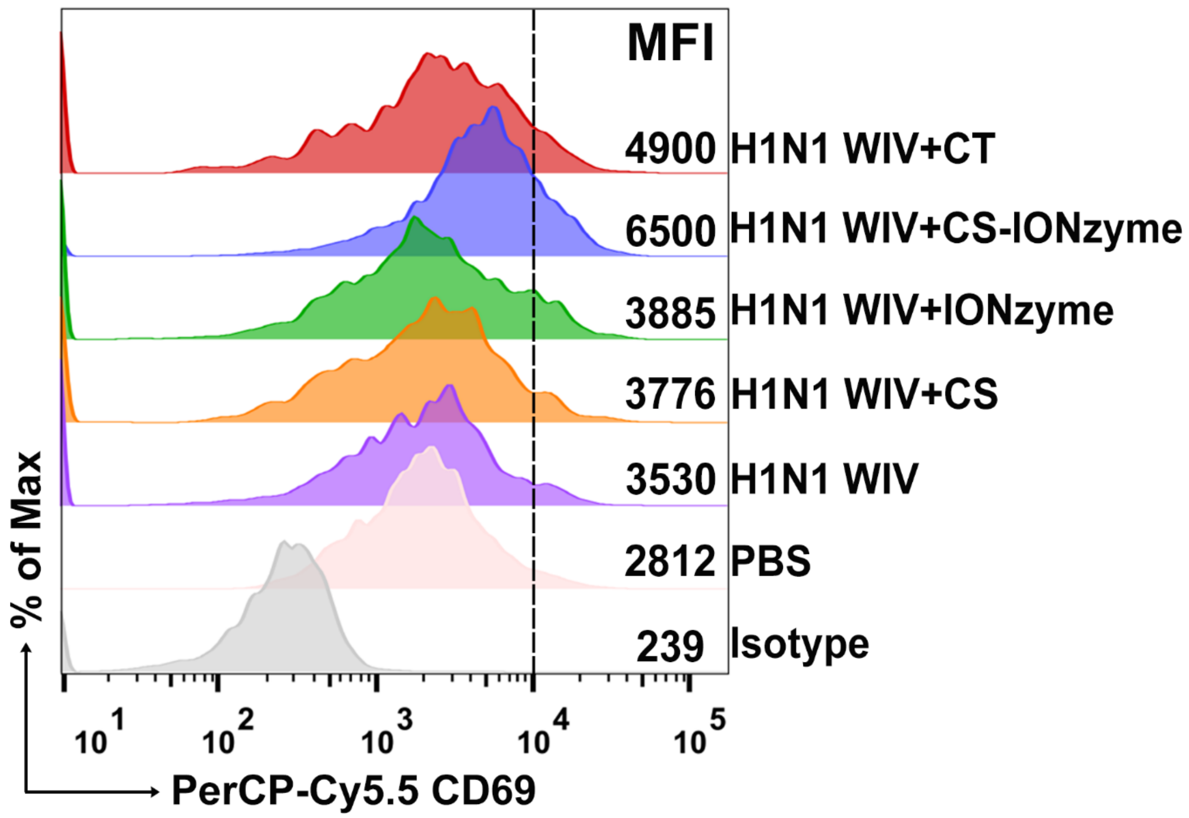

Figure S23. The expression of activation marker on splenocytes. At 28 days after the primary immunization of CS-IONzyme-based influenza vaccine in mice, splenocytes (n=6/group) were isolated and restimulated by H1N1 WIV (10 µg/mL) following 72 h *in vitro*. FCM analysis of splenocyte activation (assessed as CD69 expression). Images are from a representative experiment.

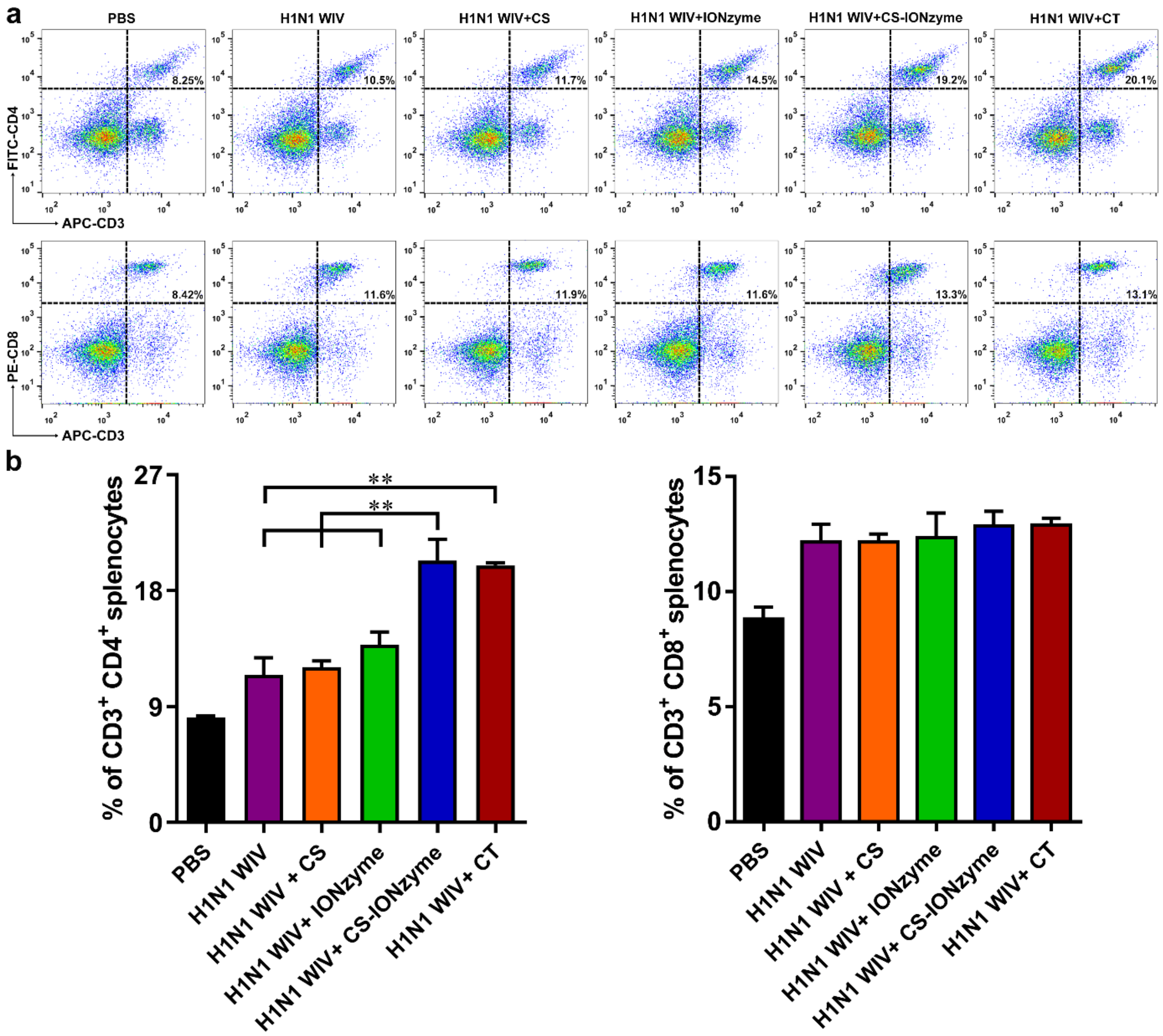

Figure S24. The percentages of $CD3^+$ $CD4^+$ and $CD3^+$ $CD8^+$ splenic T cells. At 28 days after the primary immunization of CS-IONzyme-based influenza vaccine in mice, splenocytes (n=6/group) were isolated. a) FCM analysis of the percentages of $CD3^+CD4^+$ and $CD3^+CD8^+$ splenic T cells from the immunized mice. b) Quantification of the FCM results as shown in panel a. All of the data are presented as means±s.d. and are representative of two independent experiments. Statistical significance is assessed by One-way ANOVA analysis. $^{**}P<0.01$.

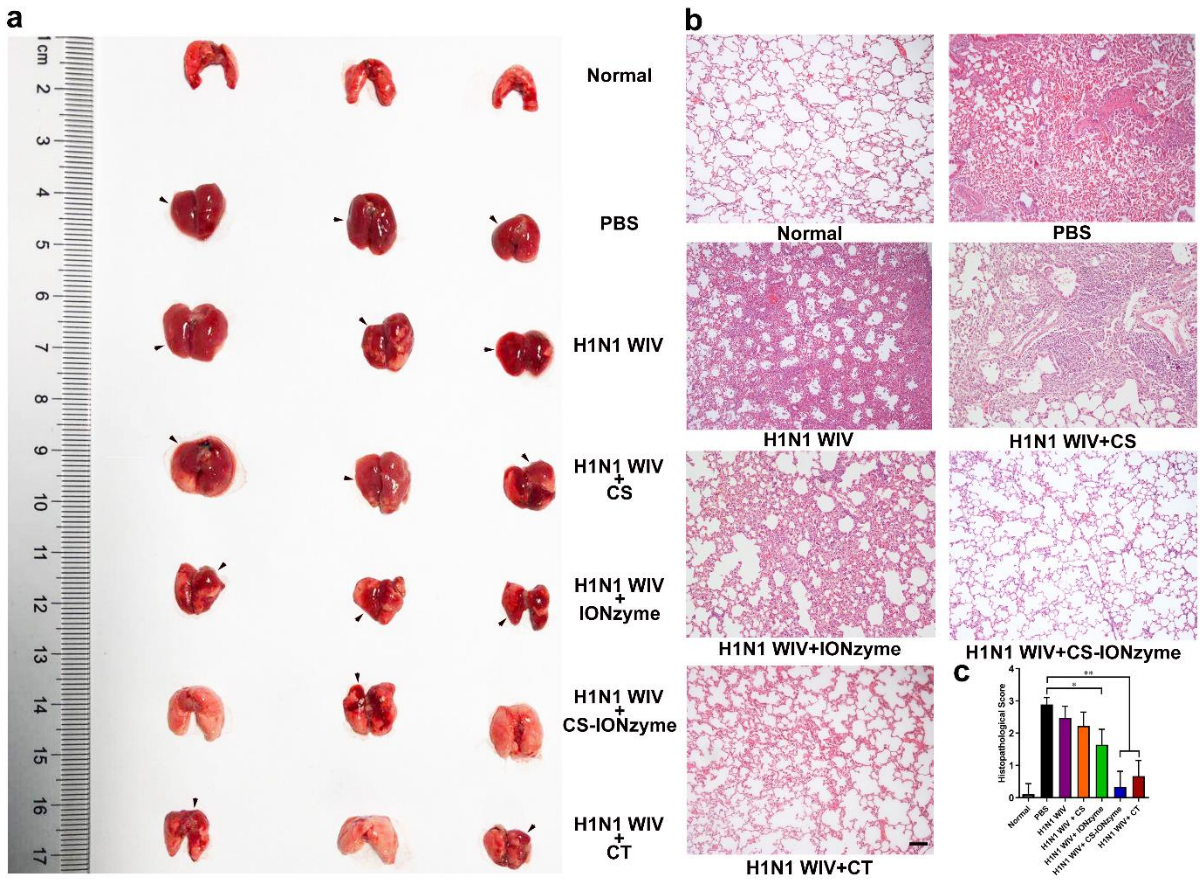

Figure S25. The pathological and histopathological change of murine lungs post challenge. The vaccinated mice were challenged with 10^6 EID_{50} of H1N1 virus 28 days post primary immunization. a) The pathological changes in lungs (n=3/group) on day 5 p. i.. b) Representative histopathological changes in H&E (hematoxylin and eosin)-stained lung tissues on day 5 p. i.. Bars: 500 μm. c) Histopathologic scores in the lungs. Data shown represent the means±s.d. Results are from one representative experiment of two performed. Statistical significance is assessed by One-way ANOVA analysis to compare the results between different groups. **P*<0.05; ***P*<0.01.

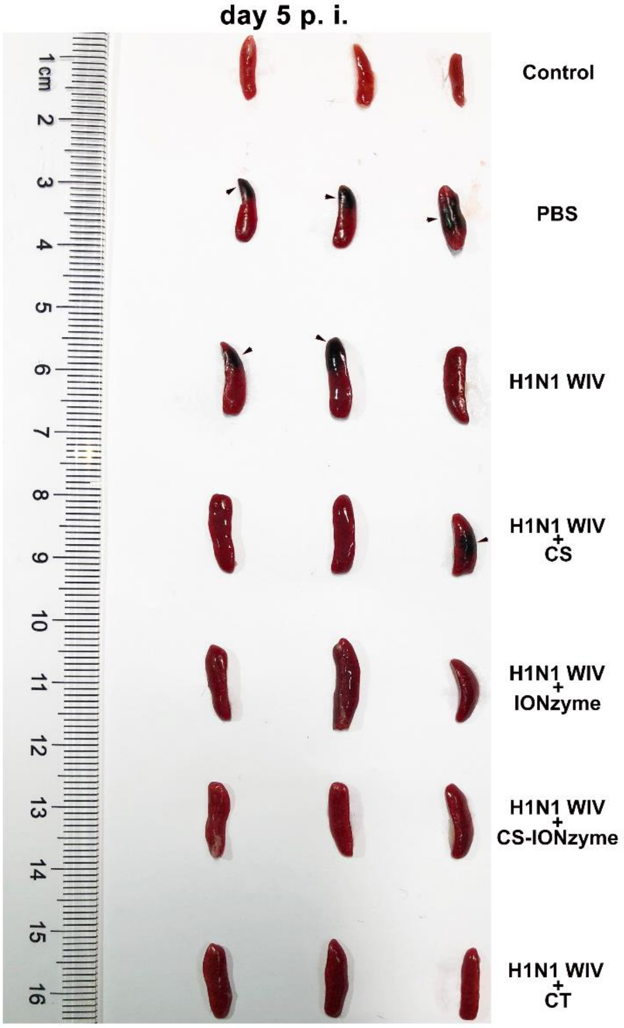

Figure S26. The pathological change of murine spleens post challenge. The vaccinated mice were challenged with 10^6 EID_{50} of H1N1 virus 28 days post primary immunization. The pathological changes in spleen (n=3/group) on day 5 p. i.. The pictures are from one representative experiment of two performed.

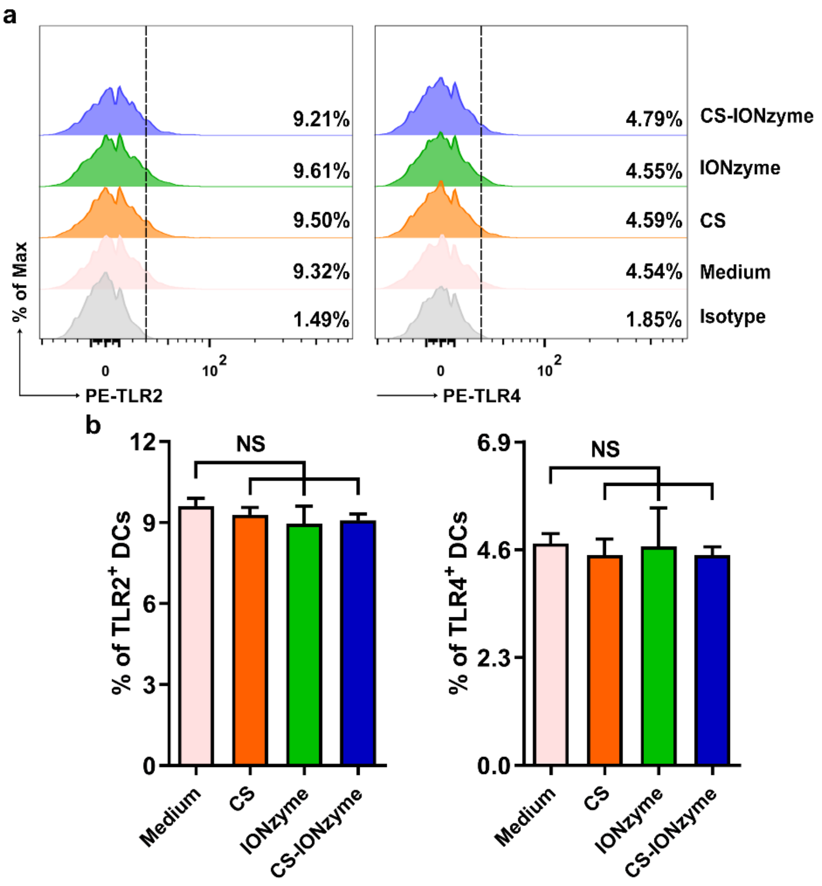

Figure S27. The expression of TLR2/4 on DCs *in vitro*. Murine DCs were treated with CS-IONzyme or other groups for 24 h. a) The expression of TLR2/4 on DCs was analyzed by FCM. Images are from a representative experiment out of three. b) Quantification of the FCM results as shown in panel a. The data are presented as means±s.d. of three replicates and are representative of three independent experiments. Statistical significance is assessed by One-way ANOVA analysis. *NS* means no significant.

Table S1 List of antibodies used for flow cytometry

Fluorescein	List of antibodies	Clone	Manufacture	Cat#
FITC	CD4	RM4-5	BD	553046
	CD45	30-F11	BD	553080
	CD86	GL1	BD	561962
	MHCII (I-A/I-E)	2G9	BD	553623
	TLR9	5G5	Abcam	ab58864
PE	CCL5 (RANTES)	2E9/CCL5	Biolegend	149104
	EPCAM (CD326)	G8.8	BD	563477
	CD45	30-F11	BD	553081
	TLR1 (CD281)	eBioTR23(TR23)	eBioscience	12-9011-80
	TLR2 (CD282)	CB225	Biolegend	148604
	TLR3 (CD283)	PaT3	BD	565984
	TLR4 (CD284)	MTS510	BD	558294
	TLR6	418601	R&D	FAB1533P
	TLR7	Polyclonal	Novus	NBP2-24761
	TLR8	44C143	Abcam	ab45097
	CD40	3/23	BD	553791
	CD80	16-10A1	BD	553769
	CCR7 (CD197)	4B12	BD	560682
	CD8α	53-6.7	BD	553033
APC	EPCAM (CD326)	G8.8	BD	563478
	CD45	30-F11	BD	561018
	CD11c	HL3	BD	550261
	CD3 (CD3e)	145-2C11	BD	553066
Alexa Fluor 647	TLR5 (CD285)	ACT5	Biolegend	148104
PerCP-Cy5.5	CD69	H1.2F3	BD	551113
	CD11c	HL3	BD	560584
Alexa Fluor 700	CCL20/MIP-3α	114906	R&D	IC760N

Table S2 List of antibodies used for immunofluorescence

Cell Marker	Primary antibody Host (source)/Dilution in PBS	Secondary antibody (source)/Dilution in PBS	Primary Antibody cat. No./clone
CD11c-Dendritic cells	Armenian hamster (Abcam)/1:100	AffiniPure Goat anti Armenian Hamster-649 (Jackson ImmunoResearch Laboratories)/1:500	ab33483/N418
CCL20/MIP-3α-Epithelial cells	Rabbit (Abcam)/1:50	Goat anti Rabbit-488 (Abcam)/1:500	ab9829/Polyclonal
